# Emotion Perception in Hadza Hunter-Gatherers

**DOI:** 10.1038/s41598-020-60257-2

**Published:** 2020-03-02

**Authors:** Maria Gendron, Katie Hoemann, Alyssa N. Crittenden, Shani Msafiri Mangola, Gregory A. Ruark, Lisa Feldman Barrett

**Affiliations:** 1 Yale University, Department of Psychology, New Haven, USA; 2 Northeastern University, Department of Psychology, Boston, USA; 3 University of Nevada, Las Vegas, Department of Anthropology, Las Vegas, USA; 4 University of Arizona, James E. Rogers College of Law, Tucson, USA; 5 U.S. Army Research Institute for the Behavioral and Social Sciences, Foundational Science Research Unit (FSRU), Fort Belvoir, USA; 6 Massachusetts General Hospital, Martinos Center for Biomedical Imaging and Department of Psychiatry, Boston, USA

**Keywords:** Evolution, Human behaviour

## Abstract

It has long been claimed that certain configurations of facial movements are universally recognized as emotional expressions because they evolved to signal emotional information in situations that posed fitness challenges for our hunting and gathering hominin ancestors. Experiments from the last decade have called this particular evolutionary hypothesis into doubt by studying emotion perception in a wider sample of small-scale societies with discovery-based research methods. We replicate these newer findings in the Hadza of Northern Tanzania; the Hadza are semi-nomadic hunters and gatherers who live in tight-knit social units and collect wild foods for a large portion of their diet, making them a particularly relevant population for testing evolutionary hypotheses about emotion. Across two studies, we found little evidence of universal emotion perception. Rather, our findings are consistent with the hypothesis that people infer emotional meaning in facial movements using emotion knowledge embrained by cultural learning.

## Introduction

It has long been claimed that certain configurations of facial movements, such as smiles, scowls, and frowns, are universally recognized as emotional expressions because they evolved to signal emotional information in situations that posed fitness challenges for our hunting and gathering hominin ancestors. This hypothesis can be traced back, in part, to Charles Darwin’s 1872 publication of *The Expression of the Emotions in Man and Animals*^1^, in which he stipulated that emotions are “expressed” across the animal kingdom via patterns of muscular discharge, such as coordinated sets of facial muscle contractions. Darwin’s hypothesis was later modified and elaborated on by evolutionary psychologists, who proposed that the facial configurations in question evolved as emotion-specific expressions to signal information^2^ in the situations our hominin ancestors faced on the African savannah during the Pleistocene^3,4^. For example, the wide-eyed gasping facial configuration, thought to universally express fear, purportedly evolved to enhance sensory sampling that supports efficient threat detection^5^, including the detection of dangerous predators. Similarly, the nose-wrinkle configuration, thought to universally express disgust, purportedly evolved to limit exposure to noxious stimuli^6^, such as food that is contaminated or that has spoiled in the heat. These hypothesized facial expressions, along with scowls (for expressing anger), smiles (for expressing happiness), and others – now over twenty, in total—are thought to be universally observed in people around the world, although slightly modified by culture^7,8^.

To test whether the facial configurations in question evolved to express certain emotion categories in a universal manner, as proposed, scientists have largely studied how people *infer* the emotional meaning of those configurations; the logic being that the production and perception of emotional expressions co-evolved as an integrated signaling system^2^ (for discussion, see^9^). This experimental approach can also be traced back to Darwin, who conducted research with two different methods to test his hypotheses^1^. Darwin first asked informants to provide their own emotion labels for photographs of the facial configurations in question. This *free-labeling* response method produced substantial variation, providing little support for Darwin’s hypotheses (see^1^, p. 12). Darwin also surveyed well-traveled colleagues and missionaries from the “old and new worlds” to learn about the facial movements of people who lived in remote, non-urbanized cultural contexts. He constrained informants’ responses by providing them with verbal descriptions of the facial configurations. Each description contained an emotion word corresponding to the category he believed was being expressed (e.g., “Does shame excite a blush when the colour of the skin allows it to be visible? and especially how low down the body does the blush extend?” p. 12). Following Darwin’s lead, modern experiments also restrict participants’ options for inferring the psychological meaning of facial configurations by having them match photos of posed facial configurations and a limited number of emotion words (with or without brief stories), a method known as *choice-from-array*^10,11^. Choice-from-array methods limit the possibility of observing cross-cultural variation that would disconfirm the hypothesis of universal facial expressions, whereas free-labeling methods allow for more discovery of cross-cultural differences^12–14^. Choice-from-array methods can even make novel emotion categories and contrived vocalizations appear universal in participants from larger, industrialized cultural contexts, in both the cultural east and west, and in a small-scale societal context^15^.

**Figure 1:**
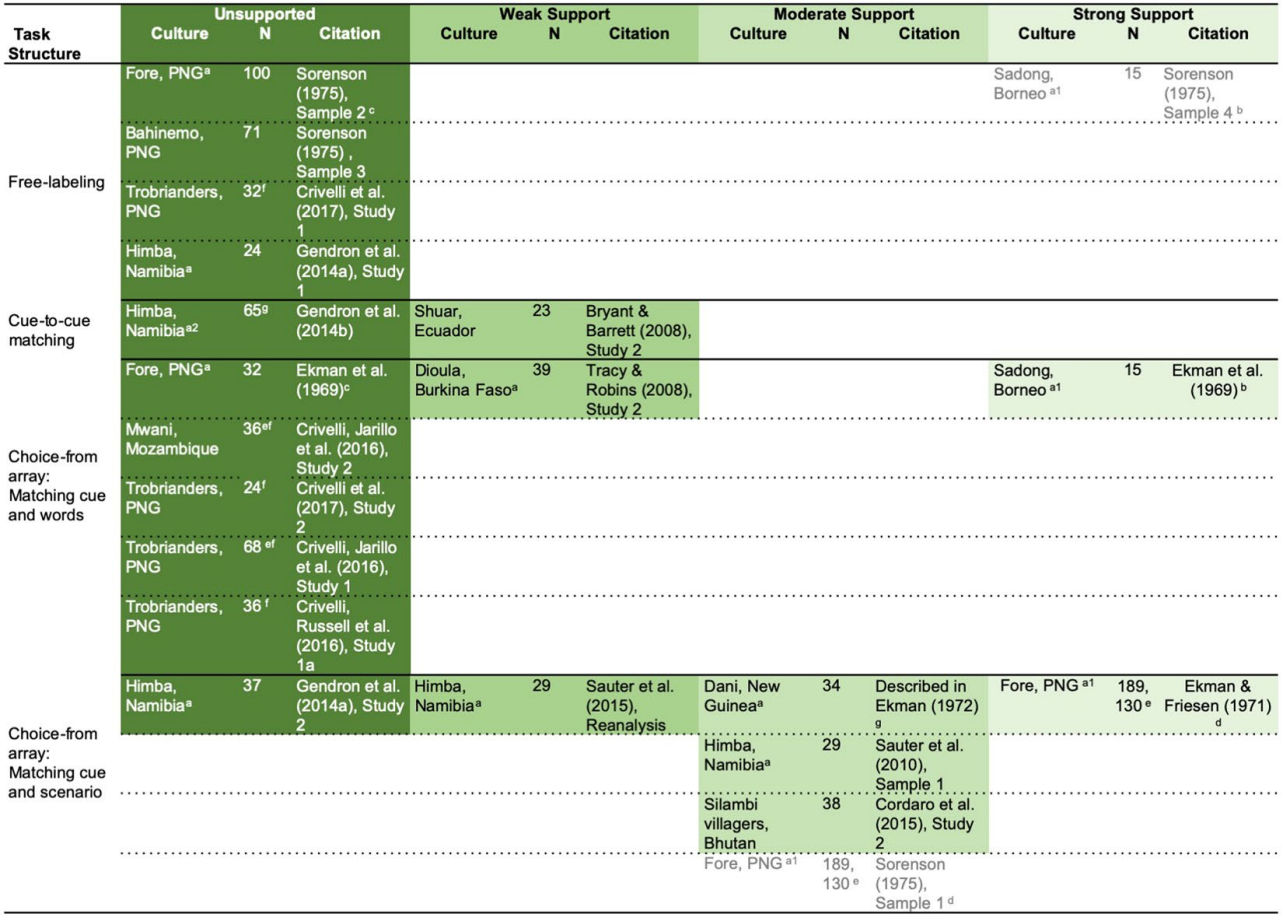
Tests of the Universality Hypothesis for Facial Configurations and Vocal Cues in Small-Scale Societies. Findings summarized for anger, disgust, fear, sadness, and surprise; happiness is the only pleasant category tested in all studies except Tracy and Robins (2008), and therefore perception can be (and likely is) guided by distinguishing valence in those studies. All studies used photographs of posed facial configurations or posed vocalizations, except Crivelli, Jarillo *et al*. (2016), Study 2, and Crivelli *et al*. (2017), Study 1, which used dynamic as well as static posed configurations and static spontaneous configurations from Papua New Guinea (PNG), respectively. In Bryant and HC Barrett (2008), participants were tested in a second language (Spanish) in which they received training. A subset of choice-from-array studies did not control whether foils and target facial configurations could be distinguished by valence and/or arousal, with the exception of Gendron *et al*. 2014a, Study 2, which controlled for valence and arousal; Sauter *et al*. 2015 (2010 re-analysis) and Cordaro *et al*. (2015) controlled for valence only. N = sample size. Unsupported = consistency and specificity at chance, or any level of consistency above chance combined with evidence of no specificity. Weak support = consistency between 20% and 40% (weak) for at least a single emotion category (other than happiness) combined with above-chance specificity for that category, or consistency between 41% and 70% (moderate) for at least a single category (other than happiness) with unknown specificity. Moderate support = consistency between 41% and 70% (moderate) combined with any evidence of above-chance specificity those categories, or consistency above 70% (strong) for at least a single category (other than happiness) with unknown specificity. Strong support = strong evidence of consistency (above 70%) and strong evidence of specificity for at least a single emotion category (other than happiness). Superscript a: Specificity levels were not reported. Superscript a1: Specificity inferred from reported results. Superscript a2: Traditional specificity and consistency tests are inappropriate for this method, but the results are placed here based on the original author’s interpretation of multidimensional scaling and clustering results. Superscript b: The sample size, marginal means, and exact pattern of errors reported for the Sadong samples is identical in Sorenson (1975), Sample 3 and Ekman *et al*. (1969); Sorenson described using a free-labeling method and Ekman *et al*. (1969) described using a choice-from-array method in which participants were shown photographs and asked to choose a label from a small list of emotion words; Ekman (1994) indicated, however, that he did not use a free-labeling method, implying that the samples are distinct. Superscript c: Sorenson (1975), Sample 2 included three groups of Fore participants (those with little, moderate, and most other-group contact). The pattern of findings is nearly identical for the subgroup with the most contact and the data reported for the Fore in Ekman *et al*. (1969); again, Sorenson described using a free-labeling method and Ekman *et al*. (1969) described using a choice-from-array method. It is questionable whether the Sadong and the Fore subgroup should be considered a small-scale society (see Sorenson, 1975, p. 362 and 363), but we include them here to avoid falsely dichotomizing cultures as “isolated from” versus “exposed to” one another (Fridlund, 1994; Gewald, 2010). Superscript d: These are likely the same sample because the sample sizes and pattern of data are identical for all emotion categories except for the fear category, which is extremely similar, and for the disgust category, which includes responses for contempt in Ekman and Friesen (1971) but was kept separate in Sorenson (1975). Superscript e: Participants were children. Superscript f: Participants were adolescents. Superscript g: The Dani sample reported in Ekman (1972) is likely a subset of the data from Ekman, Heider, Friesen, and Heider (unpublished manuscript).

Scientists largely agree that the strongest test of the universality hypotheses —e.g., that certain emotion categories and their proposed expressions were designed by natural selection to solve adaptive problems faced by our hunter-gatherer ancestors – will come from observing individuals from small-scale, non-industrialized societies^16,17^, including contemporary foraging communities. While small-scale foraging societies are neither analogs of the past nor necessarily living in remote regions untouched by outside cultural influences, they do have characteristics that make them important study populations for psychologists exploring behavioral, cognitive, or emotional phenomena. They consume a diet that is largely composed of wild foraged foods, live in highly communal and close-knit social groups, and engage in flexible residence patterning (meaning they choose who they live with) - characteristics most similar to the ecological and social contexts in which emotions and their expressions purportedly evolved. Furthermore, research with foraging communities also offers the opportunity to reveal human diversity in emotional phenomena, if present. While previous studies have explored emotion in some small-scale, non-industrial populations (summarized in Fig. 1), none have focused on foraging communities. Those studies only provide mixed evidence in support of the universality hypothesis (i.e., uniformity across societies) vs. cultural diversity (i.e., variation across societies; for review see^10^). And, notably, until 2008, *only three papers* (two of them peer reviewed) examined the perception of anger, sadness, fear, disgust, happiness, and surprise in formal experiments with such small-scale societies^8,18,19^. These were published during the period of 1969 to 1971 and included participants sampled from four small-scale, non-industrial societies in Melanesia and Southeast Asia. All used choice-from-array methods. Participants either chose a photographed facial configuration to match to a brief vignette that described an emotion category, or chose a photograph to match to an emotion word. Since 2008, a larger number of emotion perception experiments have been published (eleven so far); these studies were conducted with participants from a greater diversity of social and ecological contexts, across six small-scale societies in Africa, South America, South Asia, and Melanesia. Four studies report findings consistent with the universality hypothesis, three using a choice-from-array method^20–23^. The remaining studies, using a variety of methods (including choice-from-array, perceptual matching, and free-labeling methods) replicate one another in observing substantial variation in the meaning inferred in the facial configurations of interest^24–28^. Participants in those studies did not consistently infer the specific emotional meanings proposed by the universality hypothesis.

Yet the most compelling test of the evolutionary universality hypothesis would involve testing individuals who live in close-knit nomadic or semi-nomadic communities and collect the majority of their diet from wild foods, similar to conditions facing humans and our ancestors for most of our evolutionary history, living in similar ecosystems in sub-saharan East Africa. Existing studies have examined emotion perception in societies that hunt and gather for at least a significant portion of their diet, but not in any country on the continent of Africa (e.g., the Bahinemo of Papua New Guinea^29^), and in small-scale African societies who do not hunt and gather (the Mwani of Mozambique^24^, who are subsistence fishermen, and the Himba of Namibia^22,27,28^, who are agro-pastoralists). To date, no studies have examined emotion perception in a contemporary foraging society inhabiting any ecosystem within the continent of Africa. That is the purpose of this report.

We conducted two studies using two response methods—free-labeling (Study 1) and choice-from-array (Study 2)—to examine emotion inferences made by participants sampled from the Hadza hunter-gatherers who live in the Great Rift Valley of Northern Tanzania. They live approximately 50 kilometers south of Olduvai Gorge, which has yielded some of the earliest evidence of our hominin ancestors, whose occupation dates back more than three million years. While there is no definitive way to determine how long Hadza ancestors have occupied this region, the archeological record suggests continuous occupation for at least 50,000 years^30^. At the time of data collection in 2016, our participants were semi-nomadic, residing in small groups in temporary camps located proximally to resources, and were consuming a diet that was largely composed of wild foods – gathered plant products, honey (of stinging and stingless bees), and game animals that were hunted with bow and arrow technology. It is important to note that the Hadza are a contemporary population, and by no means models of Paleolithic life. They do continue to occupy a woodland savannah ecosystem in the Lake Eyasi Basin of Northern Tanzania that is thought to be similar to that of our Paleolithic ancestors^31^. This ecosystem contains similar types of foods that our ancestors were thought to exploit for the bulk of human evolutionary history, although it is important to note that the Hadza have supplemented their diet with some market foods over the past twenty years^32^. Research with the Hadza provides a rare opportunity for testing the hypothesis that certain facial configurations evolved in humans, while we were all hunter-gatherers, to universally express emotions.

The value of these studies is further enhanced by the fact that the Hadza way of life is rapidly under threat due to a shifting ecological and sociodemographic landscape, including loss of land (due to increased population pressure from other groups), a decline in wild foods (due to climate change and shifting boundaries of national game reserves), and increased contact with missionaries, researchers, and non-governmental organizations^33^. At the time of data collection, there were fewer than approximately 150 adults available for sampling, so we recruited for both studies at the same two bush camps. The sample for Study 1 consisted of 43 adults and the sample for Study 2 consisted of 54 adults. The majority had no formal schooling. Thirty-one participants completed both experiments. Both samples are comparable in size to other published reports testing emotion perception in small-scale societies. Prior to our visit, the Hadza had not participated in any studies of emotion perception, although they have been the subject of social cognition research more broadly^34,35^. US comparison samples included 45 and 48 adults from the community, respectively.

In Study 1, both Hadza and US participants freely labeled a set of posed facial configurations like those used in prior studies of emotion perception. These configurations are the proposed universal expressions for anger, disgust, fear, happiness, sadness, and surprise categories. We examined the extent to which participants perceived the facial configurations of interest as conveying emotional information, and to which emotion category each was assigned. This allowed us to discover whether Hadza and US participants were similar in the consistency and specificity with which they inferred emotional meaning in these configurations (supporting the universality hypothesis), or whether the Hadza were more variable in the labels they offered (i.e., an observation of cross-cultural diversity). In Study 2, participants registered their inferences using a choice-from-array response method. On a given trial, participants heard a brief story about an emotional event, including an emotion word, and then were shown two posed facial configurations and asked to choose which was the best match. We presented participants with only two facial poses to choose from, in keeping with Ekman and Friesen’s original method^19^ (where participants received 2 or 3 choices), on which our task was modeled, and consistent with more recently published studies of emotion perception in small-scale societal contexts^22,23^. In both studies, instructions and materials were presented to Hadza participants in their first language, Hadzane. In Study 1, participants responded on a given trial in Hadzane or in Swahili, their primary or second language, according to their preference. Responses were translated online, at the time of testing, into English by author SM, who is fluent in English, Swahili, and Hadzane, and entered by an experimenter. All original responses were also audio recorded so that online translations could be checked for accuracy (see Supplementary Information for details).

## Results

### Study 1: Free-labeling of facial configurations

Both Hadza (N = 43) and US (N = 45) participants were presented with six posed facial configurations in randomized order (the hypothesized expressions for anger, disgust, fear, happiness, sadness, and surprise) and were asked to freely label them. We hypothesized that when Hadza participants used mental state words to label the facial configurations, they would do so with less consistency and specificity than the US participants. Facial movements are not always understood as conveying meaning about internal, emotional states, however. People in a number of small-scale societies reportedly refrain from explicit mentalizing and, in some publications, describe an inability to infer the mental states of others because they experience other people’s minds as opaque. This phenomenon is referred to as *opacity of mind* in cultural anthropology^36^. Accordingly, we hypothesized a graded continuum of social inference, reminiscent of^37^, with descriptions of action (called *action identification*) anchoring one end, and internal states (called mental state inference or *mentalizing*) anchoring the other^38^. Action identification involves an inference of an agent and the behavior that the agent performed, whereas mentalizing involves the additional inference of an internal thought, feeling, or state to the agent. Action identifications involve a representation of *what* a person is doing (e.g., crying) and *how* she is doing it (e.g., shedding tears and vocalizing), whereas mentalizing also involves a representation of *why* the action is occurring in the first place (i.e., assigning a *mental cause* for the action; e.g., sadness). Prior research indicates that when Hadza participants are asked to assign punishment for a transgression, they are less likely to use available information about mental causes for behavior (intent)^34^, suggesting that they are less likely to mentalize. This was also true of participants from a small-scale agro-pastoralist society, the Himba of Namibia^34^. Correspondingly, Himba participants showed reduced mentalizing and increased action identification of facial configurations during an emotion perception task^28^; they freely labeled facial configurations as “crying”, “laughing”, “looking”, and so on (a finding replicated in yet another small-scale society, Trobriand Islanders^25^). Based on these findings, we hypothesized that Hadza participants would be more likely to label the facial configurations with action words rather than mental state words, when compared to US participants.

#### Mentalizing

We coded participants’ translated responses for whether their labels referred to mental states, including emotions and affective states^39,40^, as well as volitional (e.g., “intend”), cognitive (e.g., “remember”), and moral (e.g., “forgiving”) states^41^, Cohen’s Kappa for inter-coder reliability: US data κ = 1.00, Hadza data κ = 0.85 (for additional detail, see Supplementary Information text). As predicted, US participants produced a higher proportion of mental state language than did Hadza participants, *M*_*US*_ = 0.97 *SE* = 0.01, 95% *CI* [0.95, 0.99] versus *M*_*Hadza*_ = 0.70, *SE* = 0.03, 95% *CI* [0.68, 0.81], Welch’s *t*-test for unequal variances on the ranked data, *t*(79.63) = −2.22, *p* < 0.03, *D* = 0.50 (Glass’s delta). 

#### Emotion perception using emotion words

**Figure 2:**
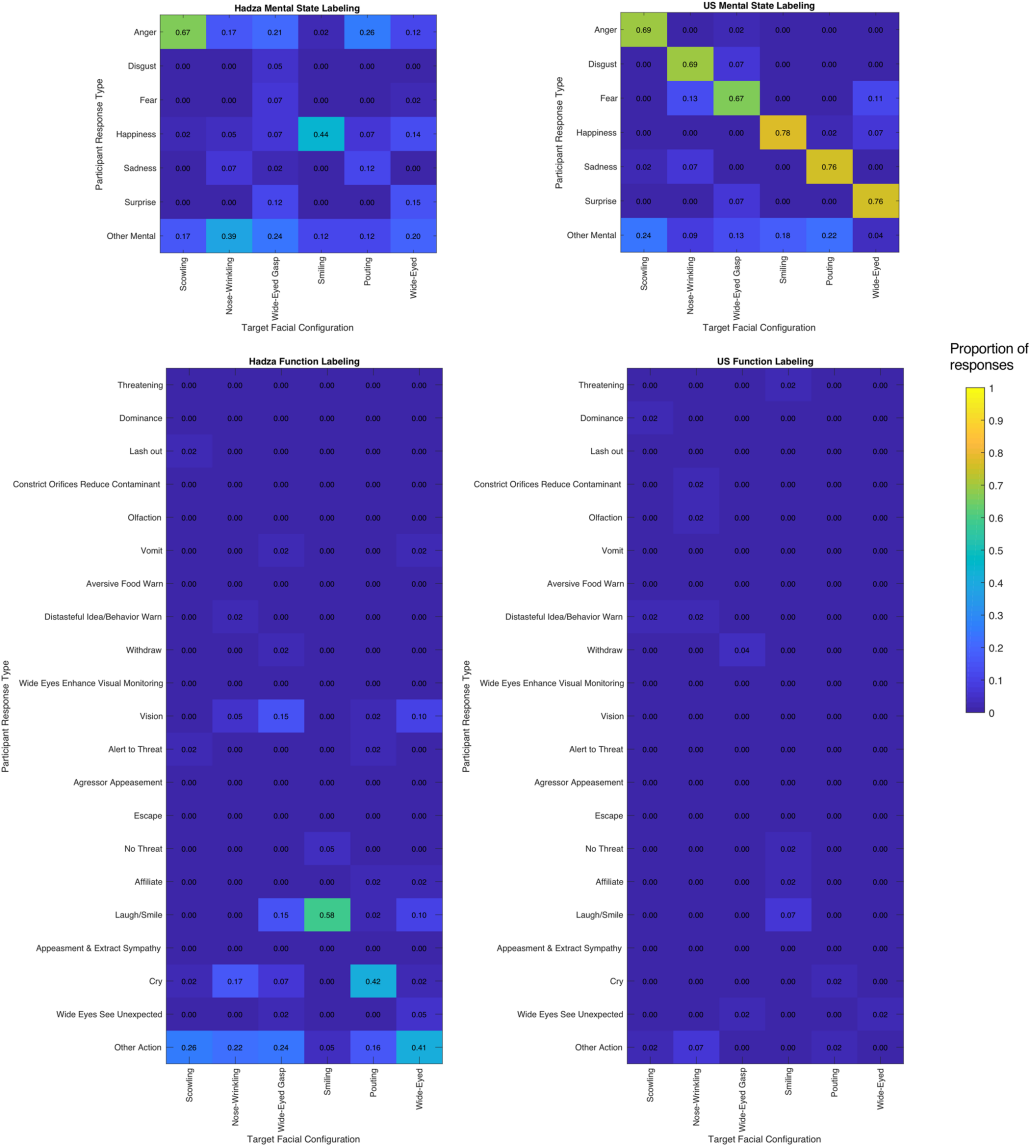
Coded responses from Study 1. Top panel depicts verbal responses produced by Hadza (left) and US (right) samples that were coded as “mental states”. The proportion of labels produced by a given sample are plotted, with higher intensity (yellow) values indicating a higher proportion and lower intensity (blue) values indicating a lower proportion; numerical proportion is also presented in each cell. Responses are plotted by the coded label types produced (y-axis) for each facial configuration of interest (x-axis). Other mental = other mental labels offered that did not conform to otherwise coded categories. Lower panel depicts verbal responses produced by Hadza (left) and US (right) samples that were coded as consistent with a set of “functional” descriptions derived from the prior literature. Functional descriptions are clustered according to their theoretically proposed links to specific emotions. Other action = other action labels offered that did not conform to otherwise coded categories.

We then coded responses for whether mental states corresponded to the emotion labels (or synonyms) associated with the universality hypothesis (*anger*, *disgust*, *fear*, *happiness*, *sadness*, and *surprise*), defined by empirically-derived semantic clusters identified for US participants^42^, Cohen’s Kappa for inter-coder reliability: US data, κ = 0.89, Hadza data, κ = 0.92. The results are presented in Fig. 2 and Table 1. US participants showed strong consistency in labeling the faces with the expected emotion words, *M*_*US*_ = 0.73, *SE* = 0.03, 95% *CI* [0.66, 0.79], when compared to Hadza participants, who displayed a weak level of agreement in providing the expected emotion labels, *M*_*Hadza*_ = 0.24, *SE* = 0.02, 95% *CI* [0.20, 0.29], Mann-Whitney test, *U* = 103, *p* < 0.001, *r* = 0.78 (weak agreement = 20% and 40%, strong agreement = above 70%, according to Haidt and Keltner^43^). This finding held for the 17 Hadza participants who spoke minimal Swahili and reported no formal schooling (which is one avenue for additional exposure to other cultural knowledge), *M*_*Hadza-M*_ = 0.25, *SE* = 0.04, 95% *CI* [0.19, 0.32].

**Table 1:** Free-Labeling Results: Study 1.

Coded Response	Facial Configurations					
	Scowl	Nose-Wrinkle	Wide-Eyed Gasp	Smile	Pout	Wide-Eyed
	Hadza (*N* = 43*)					
Anger	0.67^a^ [0.51 0.79]	0.17 [0.08 0.31]	0.21 [0.11 0.36]	0.02 [−0.01 0.13]	0.26 [0.15 0.41]	0.12 [0.05 0.25]
Disgust	0	0^b^	0.05 [0 0.17]	0	0	0
Fear	0	0	0.07^b^ [0.02 0.2]	0	0	0.02 [−0.01 0.14]
Happy	0.02 [−0.01 0.13]	0.05 [0 0.16]	0.07 [0.02 0.19]	0.44^a^ [0.3 0.59]	0.07 [0.02 0.19]	0.15 [0.07 0.29]
Sad	0	0.07 [0.02 0.19]	0.02 [−0.01 0.13]	0	0.12^b^ [0.05 0.25]	0
Surprised	0	0	0.12 [0.05 0.26]	0	0	0.15^b^ [0.07 0.29]
*χ*^2^	133.41***	19**	10.65^†^	88.60***	29.95***	14.67*
*ϕ*_*c*_	0.80	0.30	0.23	0.64	0.37	0.27
Other Mental	0.17 [0.08 0.31]	0.39 [0.26 0.54]	0.24 [0.14 0.4]	0.12 [0.05 0.25]	0.12 [0.05 0.25]	0.2 [0.1 0.34]
Lash out	0.02 [−0.01 0.13]	0	0	0	0	0
Withdraw	0	0	0.02 [−0.01 0.14]	0	0	0
Escape	0	0	0	0	0	0
Affiliate	0	0	0	0	0.02 [−0.01 0.13]	0.02 [−0.01 0.14]
Laugh/Smile	0	0	0.15 [0.07 0.29]	0.58 [0.43 0.72]	0.02 [−0.01 0.13]	0.10 [0.03 0.23]
Cry	0.02 [−0.01 0.13]	0.17 [0.08 0.32]	0.07 [0.02 0.2]	0	0.42 [0.28 0.57]	0.02 [−0.01 0.14]
*χ*^2^	4.00	35.00***	17.60*	125.00***	77.80***	12.00^†^
*ϕ*_*c*_	0.15	0.46	0.33	0.85	0.67	0.27
Other Actions	0.26 [0.15 0.41]	0.22 [0.12 0.37]	0.24 [0.14 0.4]	0.05 [0 0.16]	0.16 [0.08 0.3]	0.41 [0.28 0.57]
Constrict Orifices	0	0	0	0	0	0
Olfaction	0	0	0	0	0	0
Vomit	0	0	0.02 [−0.01 0.14]	0	0	0.02 [−0.01 0.14]
Wide Eyes/Visual Monitoring	0	0	0	0	0	0
Wide Eyes/See Unexpected	0	0	0.02 [−0.01 0.14]	0	0	0.05 [0 0.17]
Vision	0	0.05 [0 0.17]	0.15 [0.07 0.29]	0	0.02 [−0.01 0.13]	0.10 [0.03 0.23]
*χ*^2^	—	10.00	20.50***	—	5.00	12.00^†^
*ϕ*_*c*_	—	0.22	0.32	—	0.15	0.24
Threatening	0	0	0	0	0	0
Dominance	0	0	0	0	0	0
Aversive Food Warn	0	0	0	0	0	0
Distasteful Idea/Behavior Warn	0	0.02 [−0.01 0.14]	0	0	0	0
Alert to Threat	0.02 [−0.01 0.13]	0	0	0	0.02 [−0.01 0.13]	0
Aggressor Appeasement	0	0	0	0	0	0
No Threat	0	0	0	0.05 [0 0.16]	0	0
Appease/Extract Sympathy	0	0	0	0	0	0
	**US (*N* = 45)**					
Anger	0.69^a^ [0.54 0.81]	0	0.02 [−0.01 0.13]	0	0	0
Disgust	0	0.69^a^ [0.54 0.81]	0.07 [0.02 0.19]	0	0	0
Fear	0	0.13 [0.06 0.27]	0.67^a^ [0.52 0.79]	0	0	0.11 [0.04 0.24]
Happy	0	0	0	0.78^a^ [0.64 0.88]	0.02 [−0.01 0.13]	0.07 [0.02 0.19]
Sad	0.02 [−0.01 0.13]	0.07 [0.02 0.19]	0	0	0.76^a^ [0.61 0.86]	0
Surprised	0	0	0.07 [0.02 0.19]	0	0	0.76^a^ [0.61 0.86]
*χ*^2^	148.38***	110.90***	112.03***	175.00***	163.34***	128.00***
*ϕ*_*c*_	0.81	0.70	0.71	0.88	0.85	0.75
Other Mental	0.24 [0.14 0.39]	0.09 [0.03 0.21]	0.13 [0.06 0.27]	0.18 [0.09 0.32]	0.22 [0.12 0.36]	0.04 [0 0.16]
Lash out	0	0	0	0	0	0
Withdraw	0	0	0.04 [0 0.16]	0	0	0
Escape	0	0	0	0	0	0
Affiliate	0	0	0	0.02 [−0.01 0.13]	0	0
Laugh/Smile	0	0	0	0.07 [0.02 0.19]	0	0
Cry	0	0	0	0	0.02 [−0.01 0.13]	0
*χ*^2^	—	—	10.00	11.00^†^	5.00	—
*ϕ*_*c*_	—	—	0.24	0.25	0.17	—
Other Actions	0.07 [0.02 0.19]	0.04 [0 0.16]	0.07 [0.02 0.19]	0.02 [−0.01 0.13]	0.04 [0 0.16]	0.04 [0 0.16]
Constrict Orifices	0	0.02 [−0.01 0.13]	0	0	0	0
Olfaction	0	0.02 [−0.01 0.13]	0	0	0	0
Vomit	0	0	0	0	0	0
Wide Eyes/Visual Monitoring	0	0	0	0	0	0
Wide Eyes/See Unexpected	0	0	0.02 [−0.01 0.13]	0	0	0.02 [−0.01 0.13]
Vision	0	0	0	0	0	0
*χ*^2^	—	4.00	5.00	—	—	10.00
*ϕ*_*c*_	—	0.13	0.15	—	—	0.21
Threatening	0	0	0	0.02 [−0.01 0.13]	0	0
Dominance	0.02 [−0.01 0.13]	0	0	0	0	0
Aversive Food Warn	0	0	0	0	0	0
Distasteful Idea/Behavior Warn	0.02 [−0.01 0.13]	0.02 [−0.01 0.13]	0	0	0	0
Alert to Threat	0	0	0	0	0	0
Aggressor Appeasement	0	0	0	0	0	0
No Threat	0	0	0	0.02 [−0.01 0.13]	0	0
Appease/Extract Sympathy	0	0	0	0	0	0

*Note*. Proportion of coded responses provided by participants for each facial configuration in the US and Hadza samples with 95% Agresti-Coull Confidence Intervals in brackets; CI for US 0 frequency cells: [−0.02, 0.09]; CI for Hadza 0 frequency cells: [−0.02, 0.10]. Responses along the main diagonals are consistent with theoretical predictions for universal emotion or feature labeling. Cochran’s Q and McNemar pairwise comparisons were computed for the data represented along the diagonal for each cultural context separately, with superscripts indicating which responses (within culture) are statistically different from one another. *χ*^2^ goodness-of-fit tests are reported for each column, within each code type (again, within each cultural group). *p*-values are based on Monte-Carlo simulations with 10,000 replicates. Significant *χ*^2^ goodness-of-fit tests indicate that the distribution of a given response feature was not uniform across the codes for a given facial configuration. ^†^*p* ≤ 0.10. *p ≤ 0.05. ** *p* ≤ 0.01. *** *p* ≤ 0.001. Bolded proportions indicate facial configurations for which a given coded response was characteristic for that facial configuration (defined as greater than two standardized residuals based on the *χ*^2^ goodness-of-fit test; after Crivelli *et al*., 2017). Dashes indicate rows for which a *χ*^2^ test could not be computed because no responses were coded consistent with the feature. No *χ*^2^ test was computed for the social communication codes (final block of the US and Hadza portions of the table) due to low frequencies of the response feature and low reliability in coding. *Note that the the Hadza dataset contains variable number of participants across target facial configurations (N_scowl_ = 42, N_nose-wrinkle_ = 41, N_wide-eyed gasp_ = 41, N_smile_ = 43, N_pout_ = 43, N_wide-eyed_ = 41) due to a subset of responses for which a reliable translation could not be achieved (see Supplementary Information for more details).

US participants were equally consistent in freely labeling each facial configuration with the expected emotion word, Cochran’s *Q*(5) = 3.14, *p* < 0.68; see diagonals of Fig. 2 and Table 1, and their labels showed a high degree of specificity (see off-diagonals in Fig. 2 and *χ*^*2*^ goodness-of-fit tests in Table 1). Hadza participants, by contrast, labeled certain facial configurations more consistently than others, Cochran’s *Q*(5) = 67.99, *p* < 0.001, and with variable specificity (as above, see Fig. 2 and Table 1). Consistency was low and did not exceed chance-level responding for four of the six facial configurations tested.

Sixty-five percent of Hadza participants (N = 28) consistently labeled the scowling facial configuration as anger (i.e., “ofa-”), *Prop*_*Hadza*_ = 0.65, *SE* = 0.07, *p* < 0.001, 95% *CI* [0.51 0.79], which was statistically significant using a binomial test against an expected proportion of 0.16 (based on the number of available alternative facial configurations). All subsequent reported tests for above-chance consistency use this same approach. “Ofa-” was consistently applied to the scowling facial configuration at proportions well above chance, but standardized residuals of the *χ*^*2*^ tests indicated that the scowling configuration was not specifically labeled as “ofa-”: this label was also the most characteristic for the nose-wrinkle facial configuration (label offered by 7 participants), for the wide-eyed gasping configuration (label offered by 9 participants), and for the pouting configuration (label offered by 11 participants). Moreover, Hadza participants also labeled scowling faces with other terms, including the general affective description “upset” (16.70%), with action words such as “to grumble/sulk” (23.80%), or with other idiosyncratic labels (see Supplementary Information, Table 3).

Low specificity in Hadza use of the term for anger (“ofa-“) may be due to over-reliance on anger as one of the few lexicalized emotion/mental state categories^44^. When we examined the content of a dictionary compiled for the Hadza language, we counted only 21 terms that appeared to be clear references to mental states, compared to hundreds offered in the English language for the specific domain of emotion^45^. Another possibility, of course, is that Hadza participants frequently offered anger-related words because anger is actually expressed with a variety of facial configurations in Hadza culture. Instances of anger are also expressed in the US with a diversity of facial movements and low specificity of scowls to anger^12,46^, yet US participants appear to have a more narrow stereotype that they rely on (for discussion, see^12^) when compared to Hadza participants.

Forty-four percent of Hadza participants (N = 19) labeled the smiling facial configuration as an expression of happiness (“cheta” in Hadzane or “furahi” in Swahili), *Prop*_*Hadza*_ = 0.44, *SE* = 0.08, *p* < 0.001, 95% *CI* [0.30 0.59], revealing moderate consistency, even though 24 Hadza participants labeled smiling faces with other terms, including the general affective description “good” (20.90%), with action words such as “smiling” (56.00%), or with other idiosyncratic labels (see Supplementary Information, Table 3). The smiling facial configuration was labeled as happiness (“cheta/furahi”) with a statistically significant level of specificity, because “cheta/furahi” was applied to other facial configurations, but none characteristically (see Table 1). The interpretation of these findings is complicated by the fact that the smiling facial configuration was the only depiction of pleasant valence, in contrast to all the other facial configurations included in the study. As a consequence, it is unclear whether these free-labeling data support a hypothesis of universality for the emotion category of happiness or for the affective property of valence, distinguishable by zygomaticus facial muscle activation; we return to this observation when discussing a similar finding in Study 2.

#### Action identification

Responses were coded for whether they described actions such as “crying” or “seeing something”, Cohen’s Kappa for inter-coder reliability: US data κ = 0.84 Hadza data κ = 0.87. (This code was not mutually exclusive with the mental state codes reported above because full participant responses sometimes included both mental content and an action identification.) As predicted, Hadza participants labeled the facial configurations with a higher proportion of action-related labels when compared to US participants, *M*_*Hadza*_ = 0.49 *SE* = 0.04, 95% *CI* [0.41, 0.57] versus *M*_*US*_ = 0.06, *SE* = 0.02, 95% *CI* [0.03, 0.11]), Mann-Whitney *U* = 125, *p* < 0.001, *r* = 0.78.

The actions offered by Hadza participants were relatively more descriptive of actual physical movements in that they referred to *how* an agent was moving (e.g., “looking”), rather than the situational circumstances in which the actions occurred. In some cases, these action labels were situated, accompanied by details about the possible eliciting circumstances or context in which the actions occurred, but these more complex action labels were relatively less frequent. To further examine this distinction, we coded for specific *physical movements*, such as lashing out, crying, smelling, seeing, or laughing (Cohen’s Kappa range for inter-coder reliability: US data κ = 0.90–0.92, Hadza data κ = 0.79–1.00) and *social communications*, such as signaling dominance, alerting about a threat, or warning about aversive foods, using the descriptions available in^2^ (Cohen’s Kappa for inter-coder reliability: Hadza data κ = 1.00; it was not possible to compute a Kappa for US participants because they did not offer sufficient social communication responses). A full list of the codes is provided in Supplementary Information, Table 3. The results are presented in Fig. 2 and Table 1, see also Supplementary Information, Table 4. Responses that reflected social functions were extremely sparse, such that no statistical analyses of these codes could be performed.

Scientists who study emotion have a priori assigned certain actions and physiological changes to specific emotion categories^5,6,47–49^. Existing meta-analyses call these stipulations into question, however, suggesting that that actions and physiological changes are weakly consistent for, and not specific to, individual emotion categories^50,51^. Furthermore, there is no evidence that, when participants label a facial configuration with an action-related word such as “smiling” or “looking”, they are making an inference that the action is occurring in conjunction with an instance of a specific emotion category, or even during an emotional instance, per se^24–26,52^. Nonetheless, we classified the action words offered by our Hadza participants according to the cultural beliefs of western scientists and found some evidence of consistency, but only for a subset of the facial configurations, Cochran’s *Q*(5) = 64.33, *p* < 0.001. We observed that 18 participants labeled the pouting face as “crying”, which exceeded what would be expected for chance-level (0.16) consistency, *Prop*_*Hadza*_ = 0.42, *p* < 0.001, *SE* = 0.08, 95% *CI* [0.28, 0.57], but this label was also characteristic for the nose-wrinkle facial configuration, indicating poor specificity. Twenty-five participants labeled the smiling facial configuration as “laughing” or “smiling”, which exceeded what would be expected for chance-level (0.16) consistency, *Prop*_*Hadza*_ = 0.58, *p* < 0.001, *SE* = 0.08, 95% *CI* [0.43, 0.72], but this behavior was also characteristic for wide-eyed gasping (posed fear) and wide-eyed (posed surprise) targets, indicating poor specificity. US participants, by comparison, produced very few action responses and did not differ in consistency based on target facial configuration, Cochran’s *Q*(5) = 4.00, *p* < 0.549. Note that many of the responses did not conform to these categories and were more idiosyncratic in nature (see Supplementary Information, Table 5), suggesting that there were many instances in which Hadza participants did not appear to converge on a systematic description. This pattern of results implies that that some Hadza participants are unfamiliar with these facial configurations.

We also examined references to three proposed physiological functions in both Hadza and US participant responses: widening eyes to enhance vigilance, widening eyes to enhance sensory processing, and the closing of nostrils to reduce exposure to contaminants. References to these functions were sparse and lacked consistency for the proposed target facial configurations (wide-eyed gasp, wide-eyed, and nose-wrinkle; see Table 1). We also examined partial references to functions such as vision, vomit, and olfaction, even when participants did not describe the consequences of the actions (such as seeing more clearly or reduction of exposure to contaminants). Six Hadza participants made reference to vision in response to the wide-eyed gasping configuration (the proposed expression of fear), and four participants made references to vision in response to the wide-eyed configuration (the proposed expression of surprise), but neither exceeded chance-level (0.16) consistency. Descriptions of vision were characteristic for both the wide-eyed gasping and wide-eyed configuration, suggesting no specificity for a single facial configuration (see Table 1). That faces with widened eyes are described as “looking” is consistent with the hypothesis that Hadza participants may have been literally describing the facial morphology of the configurations that they viewed. This finding may also suggest that Hadza participants understood the physiological function associated with a facial movement (e.g., people see more when their eyes are widened), but does not itself imply an inference of a causal state of fear or surprise^53^.

### Study 2: Labeling facial configurations with a choice-from-array

**Figure 3:**
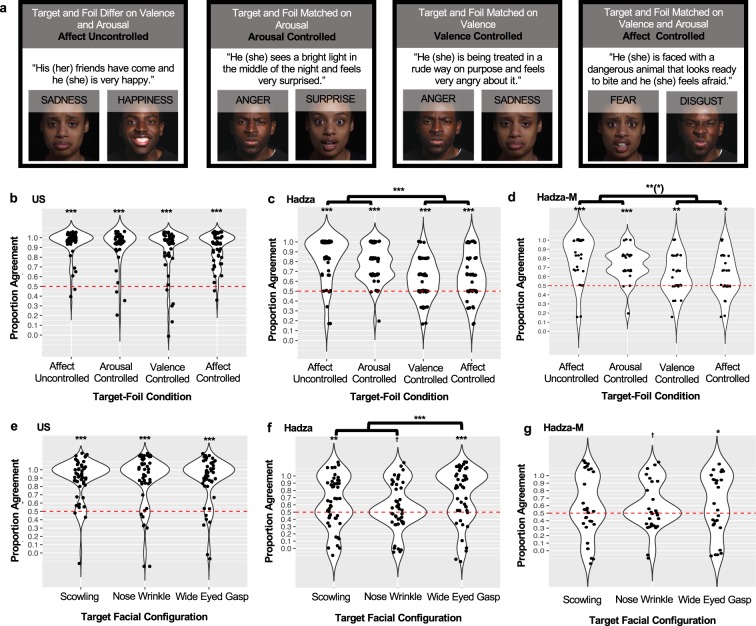
Study 2 Task Conditions (**a**) and Performance for US (**b,e**), Hadza **(c,f**) and Hadza participants with minimized exposure to other cultural groups (Hadza-M; subset based on proxy variables of second language skill and formal schooling) (**d,g**). (**a**) Examples of vignettes (for all scenarios, see Supplementary Information Table 6), targets and foils for the four trial types. Facial configurations are examples because stimulus sets restrict publication of actual photographs. *Arousal-controlled trials*: the foil face differed from the target only in depicting positivity or negativity, or valence (e.g., a smiling facial configuration hypothesized to be the universal expression of happiness vs. a scowling facial configuration hypothesized to be the universal expression of anger). Valence is a descriptive feature of affect, along with a second feature, level of arousal. For example, some evidence suggests that perceivers may be able to distinguish scowling from pouting not because scowling is perceived as “anger” and pouting is perceived as “sadness” but because scowling is typically perceived as high arousal and pouting as low arousal. *Valence-controlled trials*: the foil face differed from the target only in depicting level of arousal (e.g., a scowling vs. a pouting configuration hypothesized to be the universal expressions of anger and sadness, respectively). *Affect-uncontrolled trials:* the foil face differed from the target in depicting both valence and level arousal (e.g., a smiling vs. a pouting configuration). *Affect-controlled trials*: the foil face matched the target in depicting valence and arousal (e.g., a scowling vs. a wide-eyed gasping facial configuration hypothesized to be the universal expressions of anger and fear, respectively). Performance for each of the 4 experimental conditions (x-axis) is plotted for US participants (**b**), Hadza participants (**c**) and Hadza-M participants (**d**). Performance within the affect-controlled condition, for each of the 3 target facial configurations (x-axis) is plotted for US participants (**e**), Hadza participants (**f**) and Hadza-M participants (**g**). Individual data points represent mean proportion agreement (i.e., selecting a target matching the presumed universal model) for a given participant within a given condition. Contours of violin plots represent density of data points at a given agreement level. Horizontal red bar represents chance-level performance, and significance against chance-level responding is noted at the top of each violin plot: ***p < 0.001 **p < 0.01 *p < 0.05 ^**†**^*p* < 0.10. Means combined with brackets represent conditions that do not statistically differ in *χ*^*2*^ tests (*p*s > 0.25). Statistically significant differences between conditions based on follow-up *χ*^*2*^ tests are notated using the same conventions, with the following exception: **(*) indicates statistical significance for individual tests ranged between *p* < 0.01 and *p* < 0.001.

In Study 2, we employed a choice-from-array method, because it has provided the strongest evidence to date^19^ in support of universal perceptions of emotion from the face^10,13^,for discussion. This method only required that participants match a facial pose to an emotion word or phrase, rather than having to produce verbal labels for emotions. In addition, using this task with only two face stimuli – a target and a foil – allowed us to separately examine affect perception and emotion perception. In prior studies employing a choice-from-array method, it is possible that perceivers who appear to be distinguishing between facial poses for emotion (*emotion perception*) are merely using different affective meanings depicted by the facial configurations. For example, participants may distinguish smiling from pouting facial configurations not because smiling is perceived as “happiness” and the other configurations are perceived as “anger”, “sadness”, and so on, but because smiling is usually perceived as pleasant and pouting as unpleasant (i.e., they differ in *valence*). Prior studies in small-scale societies have documented that perceivers are able to distinguish between facial configurations that differ in the degree to which they portray pleasant vs. unpleasant states (i.e., their valence features), even as they do not consistently distinguish between the proposed facial configurations for emotion categories that are thought to be universal^24–26,28^, consonant with the hypothesis that *valence perception* is universal^54^. We designed Study 2 to distinguish valence perception from emotion perception by varying the foils that were presented to participants on each choice-from-array trial, as outlined in Fig. 3. If Hadza participants chose the expected facial configuration for a given emotion scenario on affect-controlled trials, then they must be using features other than valence and arousal to do so, providing stronger evidence for universal emotion perception. If, however, Hadza participants were less able to consistently choose the expected facial configuration for a given scenario on these *affect-controlled* trials when compared to trials where foils differed in valence features (*arousal-controlled* trials), arousal features (*valence-controlled* trials), or both features *(affect-uncontrolled* trials), then this would suggest that they are using affective features to support their performance in the task.

Note that data from a choice-from-array task, even one that strictly controls for affect perception, are still open to alternative interpretation. For example, people can use a process-of-elimination strategy when performing a forced-choice task, in which unused options from prior trials are selected^55,56^. Forced choice can also produce convergence on a label merely because it represents the best available alternative, rather than because it faithfully reflects the inference an individual is making^57^. Finally, when participants are asked to match a posed configuration of facial muscles to a brief vignette that describes a situation, they may select a target face based on contextually appropriate behavior (e.g., widening eyes when confronted with something that requires further visual attention), independent of any drawing on emotion knowledge or any process related to emotion perception.

We analyzed the choice-from-array responses using a series of nonlinear (Bernoulli) hierarchical generalized linear models in HLM7 (SSI Inc., Lincolnwood, IL) with a logit link function to estimate the log-odds that participants’ performance was above chance-level responding (i.e., selecting the hypothesized facial configuration on a given trial). We observed that both US and Hadza individuals, on average, chose the target facial configurations more often than would be expected by chance (0.5) across all four trial types (see Fig. 3b,c and Table 2). The society from which participants were sampled significantly moderated performance on all trial types (see Supplementary Information, Table 7). US and Hadza participants performed more similarly on trials in which valence features could be used to distinguish between targets and foils, consistent with the hypothesis that the perception of valence is highly replicable across societies. Hadza participants performed significantly better on trials in which valence features were available to distinguish target from foil. On affect-controlled trials, in which neither valence nor arousal could be used to distinguish target from foil, only 58% of Hadza participants selected the target facial configuration at above-chance levels (28 of 48 participants), compared to 90% who performed above chance on the arousal-controlled trials where valence features were available (43 of 48 participants). Hadza participants who had minimal other-culture exposure (based on proxy variables of formal schooling and second language fluency) had a similar pattern of performance across the four experimental conditions, although probabilities were lower (see Table 3). Most US participants (94% of the US sample), by contrast, selected the target facial configuration on the affect-controlled trials with high probability, even when valence and arousal features did not distinguish the target and foil (e.g., the scowling configuration hypothesized to be the universal expression of anger, the wide-eyed gasping expression hypothesized to be the universal expression of fear, and the nose-wrinkle configuration hypothesized to be the universal expression of disgust), suggesting that their task performance reflected inferences about emotional meaning.

**Table 2:** Choice-From Array Results: Study 2.

Model	Fixed Effect	*b*	*OR*	*CI*	*Prob*.
Hadza	Affect Uncontrolled, *β*_10_	1.414^a,^ ***	4.112	(3.057, 5.532)	0.804
	Arousal Controlled, *β*_20_	1.222^a,^ ***	3.395	(2.597, 4.438)	0.772
	Valence Controlled, *β*_30_	0.491^b,^ ***	1.634	(1.278, 2.088)	0.620
	Affect Controlled, *β*_40_	0.567^b,^ ***	1.762	(1.336, 2.325)	0.638
US	Affect Uncontrolled, *β*_10_	2.219^a,^ ***	9.198	(7.750, 10.916)	0.902
	Arousal Controlled, *β*_20_	1.936^a,^ ***	6.929	(5.475, 8.768)	0.874
	Valence Controlled, *β*_30_	1.600^b,^ ***	4.954	(3.651, 6.724)	0.832
	Affect Controlled, *β*_40_	1.922^a,^ ***	6.834	(5.217, 8.952)	0.872

*Note*. Table reports generalized linear model (HGLM) population average results with robust standard errors for Level-1 intercepts. *b* = regression coefficient, *OR* = odds ratio, *CI* = confidence interval, *Prob* = estimated probability of success. ***p ≤ 0.001. Superscripts denote whether coefficients are statistically different from one another based on *χ*^2^ hypothesis testing within a model (i.e., each society was modeled separately). Superscripts only hold for comparisons within a given society.

**Table 3:** Influence of Other-Culture Exposure in Hadza Participants: Study 2.

Fixed Effect	*b*	*OR*	*CI*	*Prob*.
**For Affect Uncontrolled slope**, ***π***_***1***_				
Minimal cultural exposure, *β*_10_	1.24***	3.44	(2.383,4.969)	0.775
Formal schooling, *β*_11_	0.01	1.01	(0.921,1.111)	
Swahili skill, *β*_12_	0.54	1.72	(0.877,3.358)	
**For Arousal Controlled slope**, ***π***_***2***_				
Minimal cultural exposure, *β*_20_	1.02***	2.76	(2.060,3.698)	0.734
Formal schooling, *β*_21_	0.02	1.02	(0.862,1.197)	
Swahili language skill, *β*_22_	0.64	1.89	(0.774,4.607)	
**For Valence Controlled slope**, ***π***_***3***_				
Minimal cultural exposure, *β*_30_	0.42**	1.52	(1.124,2.068)	0.604
Formal schooling, *β*_31_	0.045	1.05	(0.961,1.144)	
Swahili language skill, *β*_32_	−0.04	0.965	(0.530,1.752)	
**For Affect Controlled slope**, ***π***_***4***_				
Minimal cultural exposure, *β*_40_	0.43*	1.535	(1.097,2.139)	0.605
Formal schooling, *β*_41_	0.07	1.07	(0.943,1.209)	
Swahili language skill, *β*_42_	0.10	1.11	(0.600,2.045)	

*Note*. Table reports hierarchical generalized linear model (HGLM) population average results with robust standard errors for Level-1 intercepts. *b* = regression coefficient, *OR* = odds ratio, *CI* = confidence interval, *Prob* = estimated probability of success. The HGLM includes both formal schooling and Swahili language skill and as Level-2 predictors. Self-reported formal schooling was entered based on the number of years completed. Self-reported Swahili language skill was dichotomized as 0=poor, 1=good. The intercept for each condition, what we call “minimal cultural exposure”, tests performance against chance-level responding for participants who self-reported poor Swahili language skill and had no years of formal schooling. For models separately examining Schooling and Swahili as Level-2 predictors, see Supplementary Information Table 10.

#### Performance on affect-controlled trials: Emotion perception

US participants—with a probability between 0.86 and 0.89 to correctly choose the hypothesized facial configurations for anger, fear, and disgust—outperformed Hadza participants on the affect-controlled trials, which are most specific in assessing emotion perception (see Fig. 3e,f, Table 4). Hadza participants performed significantly above chance when choosing the hypothesized facial configurations for fear and anger, but not disgust (probability of correctly identifying a target on a given trial was 0.72, 0.61, and 0.59, respectively). The society from which participants were sampled significantly moderated performance for all targets (see Supplementary Information Table 8). Controlling for other-culture exposure reduced these probabilities, however, to 0.65, 0.58, and 0.60, respectively (Table 5). Of the 27 Hadza participants who spoke minimal Swahili and reported no formal schooling, 12 chose the wide-eyed gasping face for the fear category, which was significantly different from chance (see Fig. 3g, Table 5). Across all Hadza participants, level of formal schooling specifically moderated performance for the fear category; individuals with some formal schooling — involving greater exposure to cultural knowledge and norms other than their own, as well as the expectation to follow those norms — chose wide-eyed gasping facial configurations more frequently than did those with no formal schooling (see Table 5). In contrast, of the Hadza participants with minimal other-culture exposure, only nine chose the scowling facial configuration above chance for the anger category and only eight chose the nose-wrinkle facial configuration above chance for the disgust category, with the overall probabilities across participants not statistically different from chance (see Table 5).

**Table 4:** Choice-From-Array Performance in Affect-Controlled Trials: Study 2.

Model	Fixed Effect	*b*	*OR*	*CI*	*Prob*.
Hadza	Scowl, *β*_10_	0.45^a,^ **	1.57	(1.021, 2.421)	0.61
	Nose Wrinkle, *β*_20_	0.34^a, †^	1.41	(0.980, 2.031)	0.59
	Wide Eyed Gasp, *β*_30_	0.97^b,^ ***	2.63	(1.653, 4.180)	0.72
US	Scowl, *β*_10_	2.11^a,^ ***	8.21	(5.201, 12.976)	0.89
	Nose Wrinkle, *β*_20_	1.87^a,^ ***	6.51	(4.169, 10.158)	0.87
	Wide Eyed Gasp, *β*_30_	1.91^a,^ ***	6.77	(4.448, 10.317)	0.87

*Note*. Table reports generalized linear model (HGLM) population average results with robust standard errors for Level-1 intercepts. *b* = regression coefficient, *OR* = odds ratio, *CI* = confidence interval, *Prob* = estimated probability of success. ^†^*p* ≤ 0.10. **p ≤ 0.01. **p ≤ 0.001. Superscripts denote whether coefficients are statistically different from one another based on *χ*^2^ hypothesis testing within a model (i.e., each society was modeled separately). Superscripts only hold for comparisons within a given society.

**Table 5:** Influence of Other-Culture Exposure in Hadza Participants During Affect-Controlled Trials: Study 2.

Fixed Effect	*b*	*OR*	*CI*	*Prob*.
**For Scowl slope**, ***π***_***1***_				
Minimal cultural exposure, *β*_10_	0.305	1.356	(0.810,2.272)	0.576
Formal schooling, *β*_11_	0.030	1.031	(0.819,1.298)	
Swahili skill, *β*_12_	0.306	1.358	(0.379,4.858)	
**For Nose Wrinkle slope**, ***π***_***2***_				
Minimal cultural exposure, *β*_20_	0.390^†^	1.477	(0.960,2.271)	0.596
Formal schooling, *β*_21_	−0.032	0.969	(0.806,1.164)	
Swahili language skill, *β*_22_	0.018	1.019	(0.476,2.180)	
**For Wide Eyed Gasp slope**, ***π***_***3***_				
Minimal cultural exposure, *β*_40_	0.621*	1.861	(1.033,3.353)	0.650
Formal schooling, *β*_41_	0.271**	1.312	(1.072,1.605)	
Swahili language skill, *β*_42_	−0.059	0.942	(0.332,2.676)	

*Note*. Table reports hierarchical generalized linear model (HGLM) population average results with robust standard errors for Level-1 intercepts. *SE* = standard error, *df* = approximate degrees of freedom, *OR* = odds ratio, *CI* = confidence interval, *Prob* = estimated probability of success. **p ≤ 0.01. **p ≤ 0.001. The HGLM model includes both formal schooling and Swahili language skill and as Level-2 predictors. Self-reported formal schooling was entered based on the number of years completed. Self-reported Swahili language skill was dichotomized as 0=poor, 1=good. The intercept for each condition, what we call “minimal cultural exposure”, tests performance against chance-level responding for participants who self-reported poor Swahili language skill and had no years of formal schooling. For models separately examining Schooling and Swahili as Level-2 predictors, see Supplementary Information Table 11.

#### Performance in free-labeling vs. choice-from-array methods

We also examined the average proportion of agreement for the subset of participants who completed both the free-labeling and choice-from-array tasks, averaged across participants for Study 1 and averaged across trials and then participants for Study 2 (depicted in Supplementary Information Fig. 1). We adjusted the scores for guessing using a standard correction formula (proportion correct −(1/number of choices))/(1−(1/number of choices)) according to^58^. As predicted, the free-labeling method yielded lower agreement levels than did the choice-from-array method, consistent with the general pattern observed in other published studies^10–12^. These findings do not appear to be due to practice effects from Study 1 (Supplementary Information Table 9). Notably, there were no statistical differences on trials in which target and foil could not be distinguished by valence and arousal (trials that most specifically assessed emotion perception; i.e., affect-controlled trials), meaning that previously free-labeling the scowling, wide-eyed gasping, and nose-wrinkle facial configurations did not help Hadza participants to choose them as target facial configurations.

## Discussion

We conducted two studies which provided little evidence of universal emotion perception among the Hadza, a small-scale, non-industrial population of hunter-gatherers residing in Tanzania, when compared to samples drawn from the United States, a post-industrialized nation in the cultural west. Observations from our Hadza participants represent an important test of uniformity versus diversity in emotion perception, given that the window of opportunity to work among the Hadza while they are still living a predominantly foraging lifestyle is closing. Life in the Lake Eyasi basin has not remained static for the Hadza over the past century^59^ and, increasingly, environmental change is impacting foraging behaviors and mobility^60,61^. Nonetheless, research with such a community provides a rare opportunity to investigate how emotion perception among hunter-gatherers (who are semi-nomadic and reside in small groups) adds to our cross-cultural understanding of emotional phenomena. Our findings are inconsistent with hypotheses that certain facial configurations were selected as universal expressions of emotion because they may have enhanced reproductive fitness^2–4^. Instead, our findings replicate the growing number of experiments^10^ that reveal diversity, rather than uniformity, in how perceivers make sense of facial movements. Only one facial configuration – the wide-eyed gasping facial configuration – was chosen with any above-chance cross-cultural consistency in Study 2. This pattern of findings might indicate universal fear perception, were it not for the fact that this finding was neither replicated in Study 1, nor in findings from other small-scale societies^24–26^.

The present results replicate prior published findings^24,25,27^ in suggesting that people infer the valenced meaning of facial configurations similarly across societies, supporting the hypothesis that valence perception is universal^62^. Our findings also provide a possible context for reconsidering any choice-from-array studies that did not control the availability of affective features distinguishing a target face from its foil, including the landmark studies conducted by Ekman and colleagues in the late 1960s and early 1970s that have been interpreted as providing moderate to strong support for cross-cultural consistency in emotion perception^18,19,21^. Those studies may overestimate evidence in support of universality.

Our results also suggest that subtle variation in cultural exposure of the participants who were sampled in the prior literature (Fig. 1) may further account for some of the cross-cultural consistency observed in prior published findings. For example, Diola participants in Burkina-Faso^21^ were within walking distance to a town and were tested there. The Fore, a mixed subsistence population in Papua New Guinea^18,19^ resided in a protectorate of British, Germans, and Australians (between 1888 and 1975) and had sustained interactions with western settlers and missionaries; for a more detailed discussion, see^13^. Subtle variation was in evidence in the present results, with formal schooling and second-language fluency impacting the extent to which individuals conformed to the proposed universal pattern. Similar variation in emotion perception task performance based on formal schooling has been observed in the United States (e.g.,^63^). This observed impact of formal schooling on emotion perception is consistent with (although not exclusively predicted by) a constructionist hypothesis that emotion perception is enculturated (i.e., guided by learned emotion concepts that are bootstrapped into the brain during early development^64,65^). With respect to the current findings, some Hadza individuals were educated in a formal system with the historical roots in German and British colonialism in Tanzania. Individuals who attended a regional primary school received instruction in the Swahili language and were potentially also exposed to English (secondary education is conducted in the English language). In addition, individuals who attended school likely had greater exposure to individuals from other ethnic groups. As a consequence, Hadza individuals who had more formal schooling also likely had more opportunity to learn about psychological concepts, including emotion concepts, that would not have been socialized within the Hadza community.

When taken together with other published research on emotion perception in small-scale societies^10^, our findings are also consistent with the hypothesis that mentalizing is a culturally-reinforced mode of perception^66^ anchoring one end of a social inference continuum^37^. In Study 1, Hadza participants more often engaged in action identification than US participants when freely labeling the facial configurations. This pattern was observed under stringent test conditions, because in Study 1 we asked participants to freely label the facial configurations in terms of what the person was feeling, a prompt that generates robust mentalizing (and minimal action perception) in US participants. The hypothesis of a culturally-sensitive social inference continuum is consistent with prior research with members of the Himba society, who also understood facial configurations in terms of situated actions rather than in terms of inner mental states^39,40^.

Mentalizing has been assigned a number of privileged functions in social life, such as allowing humans to “predict, explain, mold, and manipulate each other’s behavior in ways that go well beyond the capabilities of other animals”^67^, p. 131. Further, in US and European psychology, mentalizing is thought to facilitate social connections and intimacy^68^, such that individuals who have difficulty with mentalizing are predicted to have deficits in social functioning. Yet even within societies that appear to reinforce a high degree of mental inference, such as the United States, there is normal variation in the extent to which people infer mental states as the cause of observable actions^38^, and this variation may be additionally driven by the individual’s goals in a given situation and relative social status^69,70^.

The present studies are not without limitations. Hadza and US participants judged only static facial configurations posed by individuals living in a western cultural context. Prior research investigating how participants from small-scale societies perceive emotion in dynamic versus static faces did not yield substantially distinct effects^24^, however, and Hadza participants labeled static poses with a variety of *dynamic behaviors*. In addition, we are unable to isolate the specific cultural features that drove differences in emotion perception between Hadza and US participants, consistent with well-documented limitations of the two-culture approach we adopted^16^. Finally, we might have observed differences in emotion perception across cultures because Hadza participants were less familiar with experimental tasks more generally. Points that mitigate this concern (in addition to our manipulation checks) include the fact that 1) the same experimental tasks have been used in published studies in other small-scale societies^19,22,27^ and, 2) the participants enrolled in our experiments were not testing naïve (both Hadza camps that we sampled from are active fieldwork sites for anthropologists and psychologists, although they had not performed emotion perception tasks prior to our testing^31,59^).

Finally, our findings are best understood as consistent with theoretical frameworks, including our constructionist account^65,71^, that hypothesize more substantial intrinsic sources of variation in both emotional expression and perception than is true for classical or prototype emotion accounts^8,72–74^ and evolutionary accounts of discrete emotions^2–4^. Recent meta-analyses and reviews indicate that instances of an emotion category such as anger, like the instances of other emotion categories, vary considerably in their associated physiological changes^75^, facial movements^76^, and even in their neural correlates, whether measured at the level of individual neurons^77,78^, as activity in specific brain regions^79^, or as distributed patterns of activity^80^. Instances of the same emotion category can vary in their affective features (e.g., some instances of fear can feel pleasant, and some instances of happiness can feel unpleasant;^81,82^). As a consequence, we propose that instances of emotion are the result of evolution, but not because they issue from innate, modular systems that promote a cascade of prepared responses, including the generation of diagnostic facial expressions. Instead, we hypothesize that instances of emotion are emergent products of multiple biologically-evolved mechanisms that depend on cultural learning^83^. We specifically propose that the developing brain bootstraps embodied concepts into its wiring, creating an internal model for how to best regulate the body across a range of situations within the constraints of a culturally-shaped world^84,85^. Accumulating evidence suggests that the human brain evolved to require an extended period of brain development, wiring itself to its physical and cultural surroundings, thereby allowing it to build a model of the world that is tailored to particular social and environmental contexts^86^. This embraining of culture may allow people to survive and thrive as a social species in a wide variety of contexts^87^. This perspective is rooted in the Darwinian concept of population thinking^65^, in which variation provides the necessary flexibility for locally-adaptive responding.

In the constructionist tradition, we hypothesize that the human brain constructs emotions, as needed, in a way that is tailored to the requirements of the immediate situation. In cognitive science, these are referred to as ad hoc categories^73^. An ad hoc category is a situated, abstract category: the instances are variable in their physical and perceptual features but similar in function, with the specific function changing from situation to situation. Consider, for example, the category for anger within western cultures: in situations involving a competition or negotiation, the anger category might be constructed such that instances share the functional goal ‘to win’^88^; in situations of threat, the anger category might cohere around the functional goal ‘to be effective’^89^ or even ‘to appear powerful’^90^; and in situations involving coordinated action, the anger category might include instances that share the functional goal ‘to be part of a group’^91^. We hypothesize that individuals learn to construct these situation-specific categories based on what is considered most functional in their immediate cultural context^15,71,92^. Correspondingly, emotional expressions may be highly variable, tailored to the demands of a situation, and may have functional forms that align with goal-directed behavior. As a result, both culturally-divergent and -convergent pathways for emotional instances, including their expressions, are predicted based on the unique and recurrent demands placed on humans across societies. Future work using a constructionist framework to guide hypothesis generation and experimental design may lead to more mechanism-based investigations of these pathways.

## Method

Both experiments were officially approved by Northeastern University’s Institutional Review Board. The research was performed in accordance with their guidelines and regulations to ensure the ethical treatment of human subjects. All participants provided informed consent before beginning the experiment. All individuals whose photos were used in the experiments consented to having their photos taken, used in scientific research and published in scientific reports. All data collection and consent procedures were approved by the Tanzanian Commission for Science and Technology (COSTECH).

### Stimuli

Pictures of posed facial portrayals of emotion used in both Study 1 and Study 2 were drawn from multiple existing stimulus sets. We selected African American targets and faces, which were further pilot tested for perceived skin pigmentation and consensus on the emotional expression portrayed (for details, see Supplementary Information text, SI Tables 1 and 2). Notably, all faces used in the main studies were rated as portraying the intended (target) emotion by at least 75% of a sample of US participants on Amazon’s Mechanical Turk. Six portrayals (one portrayal depicting *anger*, *disgust*, *fear*, *happiness*, *sadness,* and *surprise*) were employed in Study 1, and 36 portrayals depicting these same six emotions were employed in Study 2.

### Free labeling

In Study 1, 43 Hadza individuals (19 women, 24 men; ages 18–70) from two camps in the Great Rift Valley, and 45 US individuals (20 women, 25 men; ages 18–60) were presented with six different facial portrayals of emotion in random order, and were asked to freely label them (method based on^27^; details of stimulus selection and norming in Supplementary Information).

Faces were presented on a computer screen at central fixation and remained on screen until a participant provided a response. Participants were tested in a seated position, at a comfortable but not-standardized distance from the screen. Participants were allowed to move closer to the screen to inspect targets as needed. Face images were presented in rectangular photographs with an onscreen width and height of 4 × 6 inches. The faces occupied approximately 75% of the image height. Viewing distance was uncontrolled but can be estimated to vary between the lower bound of 20 inches (resulting in 11.42 × 17.06 degrees visual angle for the square photograph) and an upper bound of 40 inches (resulting in 5.72 × 8.57 degrees visual angle for the square photograph). Viewing distance was not measured during the experiment. Visual angle may thus be a nuisance variable in these data. In the field study, instructions and materials were pre-recorded in Hadzane and presented over headphones. Hadza participants responded via an interpreter either in their native language (Hadzane) or in their second language (Swahili), which is commonly spoken; responses were translated into English prior to coding. A response was coded as in agreement if participants offered a label that was semantically consistent with the expected English-language emotion category (see Supplementary Information for more details).

### Choice-from array

In Study 2, 54 Hadza individuals (25 women, 29 men; ages 18–75) from the same two camps in the Great Rift Valley were tested individually. Data from six individuals were removed prior to analysis due to non-compliance. 48 US individuals (31 women, 17 men) ranging in age from 24 to 67, with a median age of 42.50 were recruited on Mechanical Turk. Participants were required to be native English speakers, over 18 years of age, and were recruited for normal or corrected-to-normal vision. The sample included 39 individuals who identified as White, 3 individuals who identified as Black or of African descent, 1 individual who identified as Native American, and 5 individuals who reported being of mixed race or another category. Participants were recruited to have high-school level schooling or less (three of the 48 participants did not have a high school degree, and one participant had completed some college). This targeted sampling strategy was possible since our participants were recruited online (compared to Study 1, which was conducted in a public location on the Northeastern University campus).

During the experiment, a participant was presented with 24 trials, presented in a fully randomized order. On a given trial, participants listened to a short emotional vignette (that included an emotion category label; see Fig. 3a). Vignettes were adopted from the prior literature and evaluated for cultural appropriateness by two individuals (first by ANC, co-author and anthropologist who has worked among the Hadza population for 15 years, and then by co-author SM, who has long-term experience as a research assistant and is a member of the Hadza community).

In the Hadza study, task instructions and materials were presented in Hadzane. Swahili was used when no direct translation was available in the Hadzane language for specific emotion terms (i.e., “surprise” and “sadness” were translated to “shangaa” and “huzunika”, respectively). The necessity of Swahili was based on the judgment of a native speaker of Hadzane and verified via consultation of a Hadzane lexicon compiled by linguists in collaboration with native Hadzane speakers^44^. In the online US study, all task instructions were provided in written English and all vignettes were presented in audio recordings that could be played via computer speakers or headphones. In the field study, vignettes were presented over noise-cancelling headphones so that the experimenter and translator were blind to the vignette presented on a given trial.

Following the vignette, participants were asked to choose one of two pictures of posed facial expressions presented side-by-side on the computer screen. Participants indicated which face matched the emotion that the person in the vignette was feeling. Hadza participants rendered a response by pressing the face on the computer’s touch screen. US participants used their mouse to click on the target face. The face images remained on screen until a response was rendered. In both the field study in Tanzania and in the online comparison study, visual angle of the targets was not controlled. Participants were allowed to move closer to the screen to inspect targets as needed. In the field study in Tanzania, face images were presented in rectangular photographs with an onscreen width and height of 4 × 6 inches. The faces occupied approximately 75% of the image height. Viewing distance was uncontrolled but can be estimated to vary between the lower bound of 20 inches (resulting in 11.42 × 17.06 degrees visual angle for the square photograph) and an upper bound of 40 inches (resulting in 5.72 × 8.57 degrees visual angle for the square photograph). Viewing distance was not actually measured during the experiment. Participants in the United States who completed the experiment online may have even more variable visual angles since the size of the monitor on which the faces were presented was not standardized. Visual angle may thus be a nuisance variable in these data.

Each trial contained a target (based on the a priori model for universal emotions and on norming conducted in a US, English speaking sample; see Supplementary Information for details). Each trial also contained a foil that either matched the target in both valence and arousal (affect controlled), matched the target in valence but not arousal (valence controlled), matched the target in arousal but not valence (arousal controlled), or did not match the target in either valence or arousal (affect uncontrolled) (Fig. 3a).

These methods followed closely those used in Ekman and Friesen’s classic study in Papua New Guinea^19^, with two exceptions: 1) we administered the experiment on a computer with headphones, and 2) we systematically controlled the affective similarity of the foil to the target to examine cross-cultural perception of affect, as in our prior work^27^.

## Supplementary information

Supplementary Information.

## Data Availability

The de-identified datasets generated during and/or analyzed during the current study are available from the corresponding author on reasonable request. Following publication, data will be posted to the OSF by the first author.
