# Supplementary material for: Emotion Perception in Hadza Hunter-Gatherers
Source: Sci Rep. 2020 Mar 2;10:3867. doi: 10.1038/s41598-020-60257-2 (PMC7051983; doi:10.1038/s41598-020-60257-2)
Supplement: Supplementary file 1 — Supplementary Information. [file 41598_2020_60257_MOESM1_ESM.docx]

Supplementary Material For

Emotion Perception in Hadza Hunter-Gatherers

Maria Gendron^1^, Katie Hoemann^2^, Alyssa N. Crittenden^3^, Shani Msafiri Mangola^4^, Greg Ruark^5^, Lisa Feldman Barrett^2,6^

1. Yale University, Department of Psychology
2. Northeastern University, Department of Psychology

3. University of Nevada, Las Vegas, Department of Anthropology

4. University of Arizona, James E. Rogers College of Law

5. U.S. Army Research Institute for the Behavioral and Social Sciences, Foundational Science Research Unit (FSRU)

6. Massachusetts General Hospital, Martinos Center for Biomedical Imaging and Department of Psychiatry

**This document contains supporting information on the following topics:**

1. Stimulus Development: face stimulus piloting and selection
   1. Skin pigmentation pilot
   2. Emotion pilot
2. Details of testing, translation and coding of verbal responses
3. Supplementary HGLM analyses and results for Study 2
4. US Normative Ratings and FACS Codes of Face Stimuli Included in Study 1 (Table 1)
5. US Normative Ratings and FACS Codes of Face Stimuli Included in Study 2 (Table 2)
6. Coding scheme for theoretically proposed features by emotion category (Table 3)
7. Coded Response Table with stipulated features (Table 4)
8. Response Tokens in Study 1 (Table 5)
9. Scenarios used in Study 2 (Table 6)
10. HGLM Results Tables
    1. Cultural group as a Level-2 moderator of performance in the main experimental conditions (Table 7)
    2. Cultural group as a Level-2 moderator of performance within the affect controlled condition (Table 8)
    3. Hadza results results by main experimental condition with prior exposure (participation in task 1) as a level-2 predictor (Table 9)
    4. Hadza results by main experimental condition with Swahili skill and formal education level as level-2 predictors in separate models (Table 10)
    5. Hadza results by facial configuration within the Affect Controlled condition with Swahili skill and formal education level as level-2 predictors in separate models (Table 11)
11. Hadza performance in Study 1 and Study 2 (Figure 1)
12. Supplementary Information References

**I. Face stimulus piloting and selection.**

*A. Skin Pigmentation Pilot.* An initial pilot study was conducted to identify identities from existing face databases that would be most similar to Hadza faces. We asked MTurkers to rate neutral faces on degree of skin pigmentation since it is a salient dimension that distinguishes faces that the Hadza population has been previously exposed to (i.e., within group faces) from faces that are novel to the Hadza population (i.e., faces from other racial/ethnic groups to which Hadza individuals have variable levels of exposure).^1^ Face stimuli were selected from across five available stimulus sets.^2-6^ Neutral faces were piloted since they could be used to identify possible identities for use in the main experiments without the necessity of piloting all possible emotional face poses. Ratings were collected from a set of 30 individuals (16 males, 14 females; Mean age= 30.73; SD=9.68; 23 White, 3 Asian, 2 participants reported more than one race, 1 Black/African American, 1 American Indian) on Amazon’s Mechanical Turk. We also included several photographs of Hadza identities (4 male, 2 female) so that the relative degree of pigmentation of Hadza identities could be compared to the potential identities culled from the existing face databases. All Hadza faces were in the higher range of skin pigmentation among all faces rated, consistent with the findings of Little and Apicella,^1^ with Hadza identities rated between 4.5 and 5.9 on a 1 to 7 pictorial scale, with a 7 reflecting a greater degree of apparent skin pigmentation and a 1 reflecting a lesser degree of skin pigmentation. As a result, the 38 faces with the highest apparent (rated) skin pigmentation were selected for further piloting for source clarity of emotion and potential inclusion as experimental materials.

*B. Emotion Pilot.* All faces were normed and selected to have strong “source clarity” from a US cultural standpoint, following Ekman and colleagues’ guidelines ^7^. That is, we selected faces on which 75% or more of the ratings agreed that the face portrayed the target emotion. We conducted an online pilot experiment, collecting ratings from a set of 62 individuals (36 males, 26 females; Mean age= 33.5; SD=9.33) on Amazon’s Mechanical Turk. Participants rated a randomly pulled subset of 113 poses. For a given face, participants rated the extent to which (on a 1 to 5 Likert-type scale, with 1 being “not at all” and 5 being “extremely”) the face portrayed each of six emotion categories (*anger, fear, disgust, happiness, sadness, surprise*) as well as neutral affect. In addition, participants were given the option to write in an ‘other’ response. Very few write-ins were made (example responses were “tense”, “relief”, “confused”, “shock” and so on), suggesting that participants’ responses were not constrained by the rating scales. For each participant, we assigned a dominant category for a given face based on the label of the scale that was most intensely rated. These dominant category ratings were then summarized across participants to compute percent agreement with the intended portrayal. The portrayals with top agreement were selected for inclusion in Study 1 (Table 1) and Study 2 (Table 2). Final stimuli were drawn from several face sets, so they were cropped and placed against white backgrounds to create a uniform appearance across sets.

**II. Testing, translation and coding details.**

Participants were tested primarily in Hadzane, a language exclusively spoken by the Hadza which is thought to be a linguistic isolate (unrelated to languages spoken by other ethnic groups) ^8^. Many individuals in the Hadza community also speak Swahili as a second language. Bilingualism in this community likely reflects broader demographic shifts that have brought about rapid reduction in territory and a dwindling population ^9^. Translations between Hadzane and English were performed by author SM. Difficult or questionable cases were additionally reviewed by K. Miller and B. Sands, linguists with extensive knowledge of the Hadzane language. Translations from Swahili were performed by an additional field assistant, M. Peterson, who has extensive experience communicating with and translating for the Hadza, including in a research context. Details of translation and coding by task are provided below.

In Study 1, we employed a similar translation strategy. All participants were provided with pre-recorded instructions in Hadzane. Participants were allowed to provide free responses in any language of their choosing, as most individuals in the community are bilingual and frequently speak a mixture of both languages in everyday discourse. 24 participants reported moderate levels of Swahili, and 19 participants reported strong Swahili. All original responses were retained via audio recording for spot checking of translations. Some Hadzane responses were translated online, given the familiarity of our Swahili-speaking translator with some Hadzane vernacular. Some Hadzane response were translated offline by the Hadzane translator and additionally checked by two linguists who have expertise in the Hadzane language (B. Sands, K. Miller). Only a small proportion of the total data set (24 responses; 9.3%) required this intensive offline translation. Of those items, only 13 responses (5%) were unable to be resolved, due to ambiguity of the original verbal response, and were dropped from further analysis. Overall cross-cultural comparisons were computed on all remaining response data averaged (with missing trials for some participants). Additional statistical tests (emotion comparisons within culture) were performed such that participants’ data was dropped when data points were missing. In the Cochran’s Q test, six participants’ data was excluded from analysis. *N*s for individual pairwise comparisons varied accordingly. A summary of tokenized (and translated for Hadza data) responses are also provided in Table 5 for descriptive purposes.

Responses were coded by 2 trained coders (with a minimum of 20% overlap of randomly selected cases) for the following code sets: general content type (mental states, action identifications), emotion labels, and category specific feature codes (actions & physiological functions, social communications). General content codes are described in the main text. Category specific codes were based on the prior literature, as summarized in Table 3. Specific theoretical accounts of emotions as functional responses to goals that arise from recurrent environmental demands or opportunities were addressed. For example, scientists have proposed that anger is associated with an aggressive, approach oriented response. Coding of the entire dataset (including Hadza and US responses) was conducted primarily by the first author, and a random subset of the full dataset (20% of responses) were coded by the second author. These codes were largely reliable, as reported in the main text, with the exception of the Social communication code for US data which was too sparse to achieve reliability. The first author’s codes were retained for all analyses. The relevant frequencies are presented in SI Table 4 and visualized in Figure 1 of the main text.

In Study 2, participants were presented with emotion vignettes in Hadzane. Emotion words were embedded in the vignettes. When Hadzane terms were not available for a given emotion, a word from Swahili was used instead (i.e., “surprise” and “sadness” were translated to “shangaa” and “huzunika”, respectively). To gauge the appropriateness of this decision, all participants were interviewed about their comfort and familiarity with Swahili. Thirty-two participants reported moderate levels of Swahili, and 16 participants reported strong Swahili. All other task materials, including pre-recorded instructions, were provided in Hadzane by the same translator (S. Msafiri). For individuals with sufficient language skills in Swahili, the Swahili-speaking translator was used during testing. To be conservative, the Hadzane translator was used in a few instances where there were concerns regarding the ability of the participant to interact with the Swahili-speaking translator (despite the participant self-reporting moderate levels of Swahili).

**III. Details of Supplementary HGLM Analyses.**

To complement our main HGLM models (see main text), we further investigated performance within main experimental conditions and in a separate set of models, by FACIAL CONFIGURATIONwithin the affect controlled condition.

**Hadza participant characteristics as level-2 predictors.** As in the primary analyses reported in the main text, these were intercept-as-outcome model with Bernoulli trials. We were interested in the moderating effect of Level-2 participant characteristics (which serve as proxies for other-cultural exposure) on performance. In the main text, we report models that entered both Swahili language skill (dichotomized as poor=0 versus good=1) and years of formal education (in years) into the model together. This was done so that the intercept for individuals with minimal cultural exposure (as defined by both variables) could be examined. Here we report extended models that entered each of these Level-2 predictors in a separate model. Of interest was whether the slopes for these Level-2 predictors were significant, which would indicate that the performance of participants was related to education and second language fluency (both of which can be considered proxy variables for other culture-exposure). In addition, we were interested in the intercept for each of the discrete emotion conditions, which reflected the performance for the subset of participants who spoke minimal Swahili (i.e., at Swahili=0), who had no formal education (i.e., education=0). When Swahili was entered into the model, the intercept for each condition represents the subset of participants who spoke poor/moderate Swahili.

*Main Experimental Conditions.* When Swahili was entered as a Level-2 moderator, we found that the log odds of performing above chance (at .5) remained significant across all conditions. The slope of Swahili approached and met conventional levels of statistical significance for affect-uncontrolled and arousal-controlled trials, respectively (see Table 10). This finding indicates that the level of Swahili spoken by participants was related to level of performance on the experimental task, particularly on trials where valenced based information could be used to select the target. When Education was entered as a Level-2 moderator, we found that the log odds of performing above chance (at .5) remained significant across all conditions. The slope of Education approached a conventional levels of statistical significance for affect-uncontrolled trials (see Table 10). It should be noted that the moderating effect of Education and Swahili skill could be observed only in the conditions with relatively strong performance already. This finding suggests that other cultural exposure may provide a boost to performance, but only in helping participants to use affective information to distinguish between the target and foil.

*Affect-Controlled Condition.* When Swahili was entered as a Level-2 moderator, we found that the log odds of performing above chance (at .5) was significant at a conventional statistical level only for wide-eyed gasping targets (fear Facial configurations) (*p*<.05). That is, for individuals with self-reported poor Swahili skills, they only performed above-chance for fear Facial configurations. Further, for these fear FACIAL CONFIGURATIONtarget trials, the slope of Swahili approached conventional levels of statistical significance, indicating that the level of Swahili spoken by participants was related to level of performance on the experimental task (see Table 11). When Education was entered into the model, we found that the log odds of performing above chance (at .5) was significant at a conventional statistical level for fear Facial configurations and nose-wrinkling targets (disgust Facial configurations) (*p’s* <.05), but not for scowling targets (anger Facial configurations) (*p*>.05). Further, for fear target trials, the slope of Education was significant, indicating that the level of Education undertaken by participants was related to level of performance on the experimental task (see Table 11).

Table 1

*US Normative Ratings of Face Stimuli Included in Study 1*

| Emotion Portrayed | Stimulus Image Name | Stimulus Set | % Agreement | AUs Coded |
| --- | --- | --- | --- | --- |
| Anger | CFD_BM_011_020_A | CFD | 96.7 | **4E**+**7B**+**24C**+38B |
| Disgust | 39M_DI_C | McBrain | 100 | **R9E**+**R16A**+**25B** |
| Fear | CFD_BF_030_023_F | CFD | 81.5 | **1C**+**4D**+**5C**+10B+16D+21B+**25D** |
| Happy | 044_H3_007 | Gur | 100 | **12D**+**25E** |
| Sad | 032_S8_028 | Gur | 100 | **1B**+**4C**+7A+**11D**+**15D** |
| Surprise | 40M_SP_O | McBrain | 100 | **1E**+**2E**+27E |

*Note.* Original stimulus image name in source set, as well as stimulus set are noted. Percent agreement was calculated across participants in the online pilot. Bolded AUs reflect those that are proposed as characteristic for a given emotion expression.^10-13^

Table 11

*US Normative Ratings of Face Stimuli Included in Study 2*

| Emotion Portrayed | Stimulus Image Name | Stimulus Set | % Agreement | AUs Coded |
| --- | --- | --- | --- | --- |
| Anger | CFD-BF-029-046-A | CFD | 96.6 | **4D**+**7C**+12B+**17B**+**24E** |
|  | AF_ANGER | UCDSEE | 93.3 | **4E+7C**+9C+**24C** |
|  | CFD-BF-039-039-A | CFD | 93.1 | 9D+**24C** |
|  | CFD-BM-025-045-A | CFD | 92.3 | **4D**+**7B**+R15C+L15A |
|  | 43M_AN_C | McBrain | 91.7 | **4C**+**23A**+38B |
|  | CFD-BF-034-035-A | CFD | 90.9 | **4E**+**7B**+17A |
| Disgust | 40M_DI_C | McBrain | 96.7 | 4A+L10B+17B+25B |
|  | 111_D1_017 | Gur | 96 | **9E**+43D |
|  | 105_D2_023 | Gur | 93.3 | **9E**+25D+26A |
|  | 210_D21.SETZ_4 | Gur | 93.3 | **9D**+17C+25C |
|  | 045_D3_022 | Gur | 92.6 | 7C+**9E**+17D |
|  | 050_D3_019 | Gur | 92.6 | 7B+**9E**+17C |
| Fear | 43M_FE_O | McBrain | 79.2 | **1C**+**4B**+**5A**+10B+**25B** |
|  | 050_F2_015 | Gur | 78.3 | **1C**+**2D**+**5C**+10A+16C+**25D**+38A |
|  | CFD-BM-045-045-F | CFD | 77.8 | **1E**+**2D**+**4A**+**5D**+16C+**20C**+**25C** |
|  | AF_FEAR | UCDSEE | 77.3 | **1A**+**4C**+**5C**+16A+**20C**+**25A** |
|  | CFD-BF-039-022-F | CFD | 75.9 | **1B**+**4B**+6B+12D+21A+**25D** |
|  | 39M_FE_O | McBrain | 75 | **1B**+**4B**+**5C**+10C+12C+16B+**25C** |
| Happiness | 045_H2_007 | Gur | 100 | **12E**+**25E** |
|  | 050_H3_007 | Gur | 100 | **6A**+**12D**+**25C** |
|  | 111_H1_005 | Gur | 100 | 2B+**12D** |
|  | 140_H1_005 | Gur | 100 | **6A**+**12D**+**25C** |
|  | 142_H8_4 | Gur | 100 | 2A+**6B**+**12C**+**25C**+26C |
|  | 203_H1_006 | Gur | 100 | 2A+**12C**+**25A** |
| Sadness | 209_S23.SETY_4 | Gur | 100 | **1C**+**4C**+7A+**17D**+24D |
|  | AF_SADNESS | UCDSEE | 100 | 9D+**17BC** |
|  | 39M_SA_C | McBrain | 90.9 | **1A**+**4D**+7A+**15D**+**17A** |
|  | 225_S22.SETY_4 | Gur | 85.2 | **1C**+**4B**+11C+**15B**+**17B** |
|  | 045_S3_027 | Gur | 76.9 | **4B**+7A+10A+**15C**+**17C** |
|  | 153_S3_026 | Gur | 76.9 | **4D**+**11B**+**15D** |
| Surprise | F10SURP_O_ST | Barrett | 100 | **1E**+**2E**+**5C**+27C |
|  | 11F_SP_O | McBrain | 96.8 | **1E**+**2E**+**5B**+27C |
|  | AF_SURPRISE | UCDSEE | 96.7 | **1C**+4D+**5A**+**26D** |
|  | CFD-BF-037-043-F | CFD | 96.2 | **1D**+**2E**+**5B**+**25C**+**26D**+38C |
|  | F05SURP_O_ST | Barrett | 94.3 | **5D**+**26D** |
|  | CFD-BM-011-013-F | CFD | 93.8 | **2B**+**5C**+**25D**+27A |

*Note.* Original stimulus image name in source set, as well as stimulus set are noted. Percent agreement was calculated across participants in the online pilot. All stimuli exceeded 70% percent agreement on the original portrayal type intended, but the extent of agreement on a given face varied across exemplar and category. All stimuli were additionally FACS coded. Bolded AUs reflect those that are proposed as characteristic for a given emotion expression.^10-13^

Table 3

*Category Feature Coding Scheme*

|  |  |  |
| --- | --- | --- |
| Emotion | Predicted Category Content | Feature Type |
| Anger | Target Person is Impending Threat^1^ | Social Communicative Function |
|  | Communicates Dominance^1^ | Social Communicative Function |
|  | Lash out (fight, aggress, grumble)^2^ | Behavior |
| Disgust | Constricted Orifices Reduce Exposure to Contaminants^1^ | Physiological Function |
|  | Olfaction^3^ | Physiological Function (ambiguous usage) |
|  | Vomit^3^ | Physiological Function (ambiguous usage) |
|  | Warning About Aversive Foods^1^ | Social Communicative Function |
|  | Warning About Distasteful Ideas and Behaviors^1^ | Social Communicative Function |
|  | Withdraw^2^ | Behavior |
| Fear | Widened Eyes Increase Visual Field & Speed Eye Movements^1^ | Physiological Function |
|  | Vision^3^ | Physiological Function (ambiguous usage) |
|  | Alerts of Possible Threat^1^ | Social Communicative Function |
|  | Appease Potential Aggressors^1^ | Social Communicative Function |
|  | Run, Escape^2^ | Behavior |
| Happy | Communicates a Lack of Threat^1^ | Social Communicative Function |
|  | Approach, Affiliate^2^ | Behavior |
|  | Smile, Laugh^4^ | Behavior (extended) |
| Sad | Signal Appeasement & Elicit Sympathy^1^ | Social Communicative Function |
|  | Cry^2^ | Behavior |
| Surprise | Widened Eyes Increase Visual Field to See Unexpected Stimulus^1^ | Physiological Function |

*Note*. Predictions are drawn from ^1^Shariff & Tracy (2011) and ^2^Shuman et al. (2015). Several codes also expanded upon these sources: ^3^ Responses that reflect physiological processes based on those specified in Shariff & Tracy (2011), but without the requirement that the functional consequence is stated. ^4^Affiliation associated behaviors (smiling, laughing) included in a second code due to common associations in the literature.

Table 4

*Free-Label Response Coding Results for Experiment 1*

|  |  | | | | | |
| --- | --- | --- | --- | --- | --- | --- |
|  | **Facial Configurations** | | | | | |
| Coded Response | Scowl | Nose Wrinkle | Wide Eyed Gasp | Smile | Pout | Wide Eyed |
|  |  | | | | | |
|  | **US** (*n* = 45) | | | | | |
|  |  | | | | | |
| Anger | **0.69 ^a^  [**0.54 0.81] | 0 | 0.02 [-0.01 0.13] | 0 | 0 | 0 |
| Disgust | 0 | **0.69 ^a^**  [0.54 0.81] | 0.07 [0.02 0.19] | 0 | 0 | 0 |
| Fear | 0 | 0.13 [0.06 0.27] | **0.67 ^a^** [0.52 0.79] | 0 | 0 | 0.11 [0.04 0.24] |
| Happy | 0 | 0 | 0 | **0.78 ^a^** [0.64 0.88] | 0.02 [-0.01 0.13] | 0.07 [0.02 0.19] |
| Sad | 0.02 [-0.01 0.13] | 0.07 [0.02 0.19] | 0 | 0 | **0.76 ^a^** [0.61 0.86] | 0 |
| Surprised | 0 | 0 | 0.07 [0.02 0.19] | 0 | 0 | **0.76 ^a^** [0.61 0.86] |
| *χ^2^* | 148.38*** | 110.90*** | 112.03*** | 175.00*** | 163.34*** | 128.00*** |
| *φ_c_* | 0.81 | 0.70 | 0.71 | 0.88 | 0.85 | 0.75 |
| Other Mental | 0.24 [0.14 0.39] | 0.09 [0.03 0.21] | 0.13 [0.06 0.27] | 0.18 [0.09 0.32] | 0.22 [0.12 0.36] | 0.04 [0 0.16] |
| Lash out | 0 | 0 | 0 | 0 | 0 | 0 |
| Withdraw | 0 | 0 | 0.04 [0 0.16] | 0 | 0 | 0 |
| Escape | 0 | 0 | 0 | 0 | 0 | 0 |
| Affiliate | 0 | 0 | 0 | 0.02 [-0.01 0.13] | 0 | 0 |
| Laugh/Smile | 0 | 0 | 0 | **0.07** [0.02 0.19] | 0 | 0 |
| Cry | 0 | 0 | 0 | 0 | 0.02 [-0.01 0.13] | 0 |
| *χ^2^* | - | - | 10.00 | 11.00^†^ | 5.00 | - |
| *φ_c_* | - | - | 0.24 | 0.25 | 0.17 | - |
| Other Actions | 0.07  [0.02 0.19] | 0.04  [0 0.16] | 0.07  [0.02 0.19] | 0.02  [-0.01 0.13] | 0.04  [0 0.16] | 0.04  [0 0.16] |
| Constrict Orifices | 0 | 0.02 [-0.01 0.13] | 0 | 0 | 0 | 0 |
| Olfaction | 0 | 0.02 [-0.01 0.13] | 0 | 0 | 0 | 0 |
| Vomit | 0 | 0 | 0 | 0 | 0 | 0 |
| Wide Eyes/Visual Monitoring | 0 | 0 | 0 | 0 | 0 | 0 |
| Wide Eyes/See Unexpected | 0 | 0 | 0.02 [-0.01 0.13] | 0 | 0 | 0.02 [-0.01 0.13] |
| Vision | 0 | 0 | 0 | 0 | 0 | 0 |
| *χ^2^* | - | 4.00 | 5.00 | - | - | 10.00 |
| *φ_c_* | - | 0.13 | 0.15 | - | - | 0.21 |
| Threatening | 0 | 0 | 0 | 0.02 [-0.01 0.13] | 0 | 0 |
| Dominance | 0.02 [-0.01 0.13] | 0 | 0 | 0 | 0 | 0 |
| Aversive Food Warn | 0 | 0 | 0 | 0 | 0 | 0 |
| Distasteful Idea/Behavior Warn | 0.02 [-0.01 0.13] | 0.02 [-0.01 0.13] | 0 | 0 | 0 | 0 |
| Alert to Threat | 0 | 0 | 0 | 0 | 0 | 0 |
| Aggressor Appeasement | 0 | 0 | 0 | 0 | 0 | 0 |
| No Threat | 0 | 0 | 0 | 0.02 [-0.01 0.13] | 0 | 0 |
| Appease/Extract Sympathy | 0 | 0 | 0 | 0 | 0 | 0 |
|  |  |  |  |  |  |  |
|  | **Hadza** (*n* = 43*) | | | | | |
|  |  | | | | | |
| Anger | **0.67 ^a^** [0.51 0.79] | **0.17** [0.08 0.31] | **0.21** [0.11 0.36] | 0.02 [-0.01 0.13] | **0.26** [0.15 0.41] | 0.12 [0.05 0.25] |
| Disgust | 0 | 0 ^b^ | 0.05 [0 0.17] | 0 | 0 | 0 |
| Fear | 0 | 0 | 0.07 ^b^ [0.02 0.2] | 0 | 0 | 0.02 [-0.01 0.14] |
| Happy | 0.02 [-0.01 0.13] | 0.05 [0 0.16] | 0.07 [0.02 0.19] | **0.44 ^a^** [0.3 0.59] | 0.07 [0.02 0.19] | 0.15 [0.07 0.29] |
| Sad | 0 | 0.07 [0.02 0.19] | 0.02 [-0.01 0.13] | 0 | 0.12 ^b^ [0.05 0.25] | 0 |
| Surprised | 0 | 0 | 0.12 [0.05 0.26] | 0 | 0 | 0.15 ^b^ [0.07 0.29] |
| *χ^2^* | 133.41*** | 19** | 10.65^†^ | 88.60*** | 29.95*** | 14.67* |
| *φ_c_* | 0.80 | 0.30 | 0.23 | 0.64 | 0.37 | 0.27 |
| Other Mental | 0.17 [0.08 0.31] | 0.39 [0.26 0.54] | 0.24 [0.14 0.4] | 0.12 [0.05 0.25] | 0.12 [0.05 0.25] | 0.2 [0.1 0.34] |
| Lash out | 0.02 [-0.01 0.13] | 0 | 0 | 0 | 0 | 0 |
| Withdraw | 0 | 0 | 0.02 [-0.01 0.14] | 0 | 0 | 0 |
| Escape | 0 | 0 | 0 | 0 | 0 | 0 |
| Affiliate | 0 | 0 | 0 | 0 | 0.02 [-0.01 0.13] | 0.02 [-0.01 0.14] |
| Laugh/Smile | 0 | 0 | **0.15** [0.07 0.29] | **0.58** [0.43 0.72] | 0.02 [-0.01 0.13] | **0.10** [0.03 0.23] |
| Cry | 0.02 [-0.01 0.13] | **0.17** [0.08 0.32] | 0.07 [0.02 0.2] | 0 | **0.42** [0.28 0.57] | 0.02 [-0.01 0.14] |
| *χ^2^* | 4.00 | 35.00*** | 17.60* | 125.00*** | 77.80*** | 12.00^†^ |
| *φ_c_* | 0.15 | 0.46 | 0.33 | 0.85 | 0.67 | 0.27 |
| Other Actions | 0.26  [0.15 0.41] | 0.22  [0.12 0.37] | 0.24  [0.14 0.4] | 0.05  [0 0.16] | 0.16  [0.08 0.3] | 0.41  [0.28 0.57] |
| Constrict Orifices | 0 | 0 | 0 | 0 | 0 | 0 |
| Olfaction | 0 | 0 | 0 | 0 | 0 | 0 |
| Vomit | 0 | 0 | 0.02 [-0.01 0.14] | 0 | 0 | 0.02 [-0.01 0.14] |
| Wide Eyes/Visual Monitoring | 0 | 0 | 0 | 0 | 0 | 0 |
| Wide Eyes/See Unexpected | 0 | 0 | 0.02 [-0.01 0.14] | 0 | 0 | 0.05 [0 0.17] |
| Vision | 0 | 0.05 [0 0.17] | **0.15** [0.07 0.29] | 0 | 0.02 [-0.01 0.13] | **0.10** [0.03 0.23] |
| *χ^2^* | - | 10.00 | 20.50*** | - | 5.00 | 12.00^†^ |
| *φ_c_* | - | 0.22 | 0.32 | - | 0.15 | 0.24 |
| Threatening | 0 | 0 | 0 | 0 | 0 | 0 |
| Dominance | 0 | 0 | 0 | 0 | 0 | 0 |
| Aversive Food Warn | 0 | 0 | 0 | 0 | 0 | 0 |
| Distasteful Idea/Behavior Warn | 0 | 0.02 [-0.01 0.14] | 0 | 0 | 0 | 0 |
| Alert to Threat | 0.02 [-0.01 0.13] | 0 | 0 | 0 | 0.02 [-0.01 0.13] | 0 |
| Aggressor Appeasement | 0 | 0 | 0 | 0 | 0 | 0 |
| No Threat | 0 | 0 | 0 | 0.05 [0 0.16] | 0 | 0 |
| Appease/Extract Sympathy | 0 | 0 | 0 | 0 | 0 | 0 |

*Note*. Proportion of coded responses provided by participants for each facial configuration in the US and Hadza samples (with 95% Agresti-Coull Confidence Intervals in brackets; CI for US 0 frequency cells: [-0.02 0.09]; CI for Hadza 0 frequency cells: [-0.02 0.10]). Cochran’s Q and McNemar pairwise comparisons were computed for the data represented along the diagonal for each cultural context separately, with superscripts indicating which responses (within culture) are statistically different from one another. Shaded responses along the main diagonals are consistent with theoretical predictions for universal emotion or feature labeling (Blue=Emotion Labeling; Green=Behavior; Orange=Physiological Function; Yellow=Social Communications). *χ^2^* goodness-of-fit tests are reported for each column, within each code type (again, within each cultural group). P-values are based on monte-carlo simulations with 10,000 replicates. Significant *χ^2^* goodness-of-fit tests indicate that the distribution of a given response feature was not uniform across the codes for a given facial configuration. ^†^ *p*$\leq$.10. **p*$\leq$.05. ** *p*$\leq$.01. *** *p*$\leq$.001. Bolded proportions indicate facial configurations for which a given coded response was characteristic for that facial configuration (defined as greater than 2 standardized residuals based on the*χ^2^* goodness-of-fit test; after Crivelli et al., 2017). Dashes indicate rows for which a *χ^2^* test could not be computed since no responses were coded consistent with the feature. No *χ^2^* test was computed for the social communication codes (final block of the US and Hadza portions of the table) due to low frequencies of the response feature and low reliability in coding. *Note that the the Hadza dataset contains variable number of participants across target facial configurations (N_SCOWL_=42, N_NOSE WRINKLE_=41, N_WIDE EYE GASP_=41, N_SMILE_=43, N_POUT_=43, N_WIDE EYED_=41) due to a subset of responses for which a reliable translation could not be achieved (see Supplementary Information for more details).

Table 5

*Study 1 Labeling Response Tokens by Frequency of Use*

| Emotion Portrayed in Face | Hadza Labels (Translated) | US Labels |
| --- | --- | --- |
| Anger | angry (29)  grumble (10)  upset (7)  someone (2)  unhappy (2)  idiosyncratic (25) | angry (22)  frustrated (5)  upset (5)  annoyed (4)  mad (4)  focused (3)  confused (2)  idiosyncratic (21) |
| Disgust | upset (9)  angry (7)  something (6)  crying (5)  ill (4)  unhappy (4)  bad (3)  grieve (3)  happy (3)  see (3)  grumble (2)  idiosyncratic (19) | disgusted (30)  confused (4)  afraid (3)  disappointed (2)  sad (2)  scared (2)  shocked (2)  idiosyncratic (18) |
| Fear | angry (9)  something (8)  bad (7)  laughing (7)  see (6)  upset (6)  surprised (5)  happy (4)  afraid (3)  crying (3)  thinking (3)  good appearance (2)  IDK (2)  ill (2)  questioning (2)  idiosyncratic (23) | scared (14)  afraid (9)  nervous (4)  shocked (4)  disgusted (3)  startled (3)  surprised (3)  concerned (2)  grossed out (2)  worried (2)  idiosyncratic (21) |
| Happiness | laughing (26)  happy (19)  good (6)  angry (2)  no problem (2)  idiosyncratic (17) | happy (34)  confident (3)  pleased (3)  conniving (2)  content (2)  something (2)  idiosyncratic (29) |
| Sadness | crying (18)  angry (10)  questioning (4)  bad (3)  grieve (3)  grumble (3)  happy (3)  sad (2)  see (2)  smiling (2)  idiosyncratic (26) | sad (33)  upset (8)  disappointed (4)  crying (2)  depressed (2)  distraught (2)  idiosyncratic (20) |
| Surprise | surprised (6)  bad (6)  happy (6)  open mouth (6)  angry (5)  see (5)  questioning (4)  laughing (4)  ill (3)  good (3)  thinking (3)  awesome (2)  kena (2)*  dead (2)  something (2)  upset (2)  idiosyncratic (34) | surprised (33)  excited (6)  shocked (6)  fake (3)  good (2)  idiosyncratic (12) |

*Note*. *Consistent response provided for which no reliable translation could be achieved. Idiosyncratic responses are those that occurred only once in response to a given facial expression.

Table 6

*Vignettes Used in Experiment 2*

| Emotion | Story |
| --- | --- |
| Anger | He (she) is being treated in a rude way, on purpose, and feels angry about it. (s) |
| Disgust | Someone has just eaten rotten food and feels very disgusted. (s) |
| Happiness | His (her) friends have come, and he (she) is very happy. (e) |
| Fear | He (she) is faced with a dangerous animal that looks ready to bite and he (she) feels afraid. (s + e) |
| Sadness | His (her) child has died, and he (she) feels very sad. (e) |
| Surprise | He (she) sees a bright light in the middle of the night and is very surprised. (s) |

*Note.* (s) denotes vignettes that were sourced from Sauter et al. ^14^; (e) denotes vignettes that were sourced from Ekman & Friesen ^15^; (s + e) denotes vignettes that were sourced from both.

Table 7

*Influence of Society on Choice-From-Array Performance: Study 2*

| Fixed Effect | *b* | OR | CI |
| --- | --- | --- | --- |
| For Affect Uncontrolled slope, *π_1_* | |  |  |
| Effect of Society, *β_11_* | 0.77^a,^ *** | 2.17 | (1.53,3.07) |
| For Arousal Controlled slope, *π_4_* | |  |  |
| Effect of Society, *β_41_* | 1.08^a,^ *** | 2.95 | (1.94,4.50) |
| For Valence Controlled slope, *π_3_* | |  |  |
| Effect of Society, *β_31_* | 1.30^b,^ *** | 3.68 | (2.33,5.83) |
| For Affect Controlled slope, *π_2_* | |  |  |
| Effect of Society, *β_21_* | 1.40^b,^ *** | 4.05 | (2.65,6.17) |

*Note*. Table reports population average results (computed with robust standard errors). This slopes-as-outcomes model examined the impact of society (US versus Hadza) on performance within each of the main experimental conditions. *** *p*$\leq$.001. Superscripts denote whether slopes are statistically different from one another based on χ^2^ hypothesis testing (*p*’s<.05).

Table 8

*Influence of Society on Performance in the Affect Controlled Condition: Study 2*

| Fixed Effect | *b* | OR | CI |
| --- | --- | --- | --- |
| For Scowl slope, *π_1_* | |  |  |
| Effect of Society, *β_11_* | 1.64^a,^ *** | 5.16 | (2.654,10.043) |
| For Nose Wrinkle slope, *π_4_* | |  |  |
| Effect of Society, *β_41_* | 1.59^a,^ *** | 4.90 | (2.449,9.800) |
| For Wide Eyed Gasp slope, *π_3_* | |  |  |
| Effect of Society, *β_31_* | 0.96^b,^ ** | 2.62 | (1.377,4.984) |

*Note*. Table reports population average results (computed with robust standard errors). This slopes-as-outcomes model examined the impact of society (US versus Hadza) on performance for the three target Facial configurations in the Affect Controlled condition. *** *p*$\leq$.001. Superscripts denote whether slopes are statistically different from one another based on χ^2^ hypothesis testing (*p*’s<.05).

Table 9

*Influence of Prior Task Exposure on Hadza Choice-from-Array Performance: Study 2*

| Model | Fixed Effect | *b* | *OR* | *CI* | *Prob.* |
| --- | --- | --- | --- | --- | --- |
| 1 | For Affect Uncontrolled slope, *π_1_* |  |  |  |  |
|  | No Prior Exposure Sub-sample, *β_10_* | 1.88*** | 6.55 | (4.701, 9.133) | 0.87 |
|  | Prior Task Exposure, *β_11_* | -0.67** | 0.51 | (0.302, 0.859) |  |
|  | For Arousal Controlled slope, *π_2_* |  |  |  |  |
|  | No Prior Exposure Sub-sample, *β_20_* | 1.53*** | 4.64 | (2.872, 7.485) | 0.82 |
|  | Prior Task Exposure, *β_21_* | -0.46 | 0.63 | (0.353, 1.119) |  |
|  | For Valence Controlled slope, *π_3_* |  |  |  |  |
|  | No Prior Exposure Sub-sample, *β_30_* | 0.78*** | 2.19 | (1.387, 3.445) | 0.69 |
|  | Prior Task Exposure, *β_31_* | -0.44 | 0.64 | (0.377, 1.096) |  |
|  | For Affect Controlled slope, *π_4_* |  |  |  |  |
|  | No Prior Exposure Sub-sample, *β_40_* | 0.65** | 1.91 | (1.273, 2.870) | 0.66 |
|  | Prior Task Exposure, *β_41_* | -0.12 | 0.88 | (0.511, 1.527) |  |

*Note*. Table reports population average results (computed with robust standard errors). The intercept tests performance of participants who did not complete the free labeling task against chance-level responding and the effect of prior exposure is tested within each target-foil condition. **p*$\leq$.05. ** *p*$\leq$.01. ** *p*$\leq$.001.

Table 10

*Influence of Other-Culture Exposure Variables in Hadza Participants on Choice-from-Array Performance: Study 2*

| Model | Fixed Effect | *b* | *OR* | *CI* | *Prob.* |
| --- | --- | --- | --- | --- | --- |
| 1 | For Affect Uncontrolled slope, *π_1_* |  |  |  |  |
|  | Minimal Swahili Sub-Sample, *β_10_* | 1.24*** | 3.46 | (2.404,4.992) | 0.78 |
|  | Swahili Language Skill, *β_11_* | 0.59^†^ | 1.81 | (0.979,3.348) |  |
|  | For Arousal Controlled slope, *π_2_* |  |  |  |  |
|  | Minimal Swahili Sub-Sample, *β_20_* | 1.02*** | 2.78 | (2.087,3.690) | 0.74 |
|  | Swahili Language Skill, *β_21_* | 0.70* | 2.02 | (1.031,3.969) |  |
|  | For Valence Controlled slope, *π_3_* |  |  |  |  |
|  | Minimal Swahili Sub-Sample, *β_30_* | 0.44** | 1.55 | (1.146,2.091) | 0.61 |
|  | Swahili Language Skill, *β_31_* | 0.16 | 1.18 | (0.702,1.975) |  |
|  | For Affect Controlled slope, *π_4_* |  |  |  |  |
|  | Minimal Swahili Sub-Sample, *β_40_* | 0.45** | 1.56 | (1.120,2.186) | 0.61 |
|  | Swahili Language Skill, *β_41_* | 0.37 | 1.45 | (0.811,2.606) |  |
| 2 | For Affect Uncontrolled slope, *π_1_* |  |  |  |  |
|  | No Formal Ed. Sub-Sample, *β_10_* | 1.29*** | 3.63 | (2.567,5.125) | 0.78 |
|  | Formal Education, *β_11_* | 0.08^†^ | 1.08 | (0.988,1.190) |  |
|  | For Arousal Controlled slope, *π_2_* |  |  |  |  |
|  | No Formal Ed. Sub-Sample, *β_20_* | 1.07*** | 2.91 | (2.166,3.906) | 0.74 |
|  | Formal Education, *β_21_* | 0.10 | 1.10 | (0.978,1.244) |  |
|  | For Valence Controlled slope, *π_3_* |  |  |  |  |
|  | No Formal Ed. Sub-Sample, *β_30_* | 0.42** | 1.52 | (1.139,2.022) | 0.60 |
|  | Formal Education, *β_31_* | 0.04 | 1.04 | (0.963,1.131) |  |
|  | For Affect Controlled slope, *π_3_* |  |  |  |  |
|  | No Formal Ed. Sub-Sample, *β_40_* | 0.44** | 1.55 | (1.148,2.089) | 0.61 |
|  | Formal Education, *β_41_* | 0.08 | 1.08 | (0.969,1.208) |  |

*Note*. Table reports population average results (computed with robust standard errors). Model 1 includes self-reported Swahili language skill (dichotomized as 0=poor, 1=good) as a Level-2 (participant) predictor. The intercept for Model 1 tests performance of participants with self-reported poor Swahili language skill against chance-level responding. Model 2 includes education (in number of years) as a Level-2 (participant) predictor. The intercept for Model 2 tests performance against chance level responding for participants with 0 years of formal education. ^†^ *p*$\leq$.10. **p*$\leq$.05. ** *p*$\leq$.01. ** *p*$\leq$.001.

Table 11

*Influence of Other-Culture Exposure Variable in Hadza Participants on Performance in the Affect Controlled Condition: Study 2*

| Model | Fixed Effect | *b* | *SE* | *OR* | *CI* | *Prob.* |
| --- | --- | --- | --- | --- | --- | --- |
| 1 | For Scowl slope, *π_1_* |  |  |  |  |  |
|  | Minimal Swahili Sub-Sample, *β_10_* | 0.315 | 0.256 | 1.370 | (0.818,2.296) | 0.578 |
|  | Swahili Language Skill, *β_11_* | 0.434 | 0.467 | 1.543 | (0.603,3.948) |  |
|  | For Nose Wrinkle slope, *π_2_* |  |  |  |  |  |
|  | Minimal Swahili Sub-Sample, *β_20_* | 0.379^†^ | 0.213 | 1.461 | (0.952,2.243) | 0.594 |
|  | Swahili Language Skill, *β_21_* | -0.114 | 0.401 | 0.893 | (0.398,2.000) |  |
|  | For Wide Eyed Gasp slope, *π_3_* |  |  |  |  |  |
|  | Minimal Swahili Sub-Sample, *β_30_* | 0.705* | 0.291 | 2.024 | (1.126,3.638) | 0.669 |
|  | Swahili Language Skill, *β_31_* | 0.892^†^ | 0.450 | 2.439 | (0.985,6.037) |  |
| 2 | For Scowl slope, *π_1_* |  |  |  |  |  |
|  | No Formal Ed. Sub-Sample, *β_10_* | 0.338 | 0.240 | 1.402 | (0.865,2.274) | 0.584 |
|  | Formal Education, *β_11_* | 0.069 | 0.083 | 1.071 | (0.906,1.267) |  |
|  | For Nose Wrinkle slope, *π_2_* |  |  |  |  |  |
|  | No Formal Ed. Sub-Sample, *β_20_* | 0.392* | 0.195 | 1.480 | (1.000,2.191) | 0.597 |
|  | Formal Education, *β_21_* | -0.029 | 0.081 | 0.971 | (0.824,1.144) |  |
|  | For Wide Eyed Gasp slope, *π_3_* |  |  |  |  |  |
|  | No Formal Ed. Sub-Sample, *β_30_* | 0.613* | 0.269 | 1.845 | (1.074,3.171) | 0.649 |
|  | Formal Education, *β_31_* | 0.267** | 0.088 | 1.306 | (1.094,1.560) |  |

*Note*. Table reports population average results with robust standard errors. Model 1 includes self-reported Swahili language skill (dichotomized as 0=poor, 1=good) as a Level-2 (participant) predictor. The intercept for Model 1 tests performance of participants with self-reported poor Swahili language skill against chance-level responding. Model 2 includes education (in number of years) as a Level-2 (participant) predictor. The intercept for Model 2 tests performance against chance level responding for participants with 0 years of formal education. ^†^ *p*$\leq$.10. **p*$\leq$.05. ** *p*$\leq$.01. ** *p*$\leq$.001


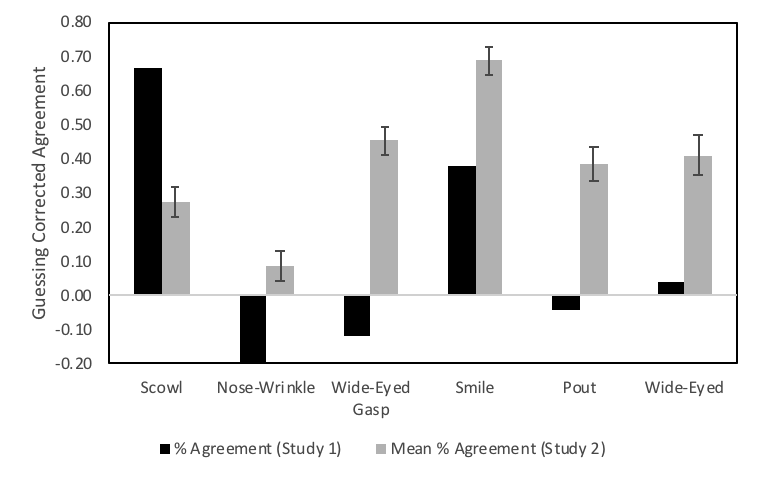


*Figure 1.* Hadza participant performance across labeling (Study1) and choice-from-array (Study 2) tasks. Guessing corrected agreement values represent adjusted mean agreement based on the number of (theoretical) response selections available. In Study 1, we corrected based on six possible responses (one for each facial configuration included in the task). In Study 2, we corrected based on the number of options present on a given trial (2: target and foil). This method was implemented after Elfenbein & Ambady (2002). Note that this method does not address response bias, a procedure which would require comparable confusion matrices across studies, which are not available due to the target-foil manipulation in Study 2. Overall, we can observe that the choice-from-array procedure from Study 2 yielded higher agreement across all categories except anger, compared to the emotion labeling method from Study 1.

**References**

1 Little, A. C. & Apicella, C. L. Face adaptation in an isolated population of African hunter-gatherers: Exposure influences perception of other-ethnicity faces more than own-ethnicity faces. *Psychonomic bulletin & review* **23**, 439-444 (2016).

2 Tottenham, N. *et al.* The NimStim set of facial expressions: judgments from untrained research participants. *Psychiatry research* **168**, 242-249 (2009).

3 Gur, R. C. *et al.* A method for obtaining 3-dimensional facial expressions and its standardization for use in neurocognitive studies. *Journal of neuroscience methods* **115**, 137-143 (2002).

4 Tracy, J. L., Robins, R. W. & Schriber, R. A. Development of a FACS-verified set of basic and self-conscious emotion expressions. *Emotion* **9**, 554 (2009).

5 Ma, D. S., Correll, J. & Wittenbrink, B. The Chicago face database: A free stimulus set of faces and norming data. *Behavior research methods* **47**, 1122-1135 (2015).

6 Barrett, L. F. (unpublished).

7 Ekman, P., Friesen, W. V. & Ellsworth, P. *Emotion in the Human Face: Guide-lines for Research and an Integration of Findings: Guidelines for Research and an Integration of Findings*. (Pergamon, 1972).

8 Sands, B. in *Language, Identity, and Conceptualization among the Khoisan* (ed Mathias Schladt) 265-283 (Rudiger Kupper Verlag, Köln, Germany, 1998).

9 Jones, N. B. *Demography and Evolutionary Ecology of Hadza Hunter-Gatherers*. Vol. 71 (Cambridge University Press, 2016).

10 Matsumoto, D., Keltner, D., Shiota, M. N., O’Sullivan, M. & Frank, M. Facial expressions of emotion. *Handbook of emotions* **3**, 211-234 (2008).

11 Du, S., Tao, Y. & Martinez, A. M. Compound facial expressions of emotion. *Proceedings of the National Academy of Sciences* **111**, E1454-E1462, doi:10.1073/pnas.1322355111 (2014).

12 Cordaro, D. T. *et al.* Universals and cultural variations in 22 emotional expressions across five cultures. *Emotion* **18**, 75-93, doi:10.1037/emo0000302 (2018).

13 Keltner, D. & Cordaro, D. T. in *Emotion Researcher* 9-24 (2015).

14 Sauter, D. A., Eisner, F., Ekman, P. & Scott, S. K. Cross-cultural recognition of basic emotions through nonverbal emotional vocalizations. *Proceedings of the National Academy of Sciences* **107**, 2408-2412 (2010).

15 Ekman, P. & Friesen, W. V. Constants across cultures in the face and emotion. *Journal of Personality and Social Psychology* **17**, 124-129 (1971).
